# Aggregate assembly of ferrocene functionalized indium-oxo clusters†

**DOI:** 10.1039/d3sc05824g

**Published:** 2023-12-04

**Authors:** Rong Zhang, Jiajing Lan, Fei Wang, Shumei Chen, Jian Zhang

**Affiliations:** a State Key Laboratory of Structural Chemistry, Fujian Institute of Research on the Structure of Matter, Chinese Academy of Sciences Fuzhou Fujian 350002 People's Republic of China wangfei04@fjirsm.ac.cn zhj@fjirsm.ac.cn; b College of Chemistry, Fuzhou University Fuzhou Fujian 350108 People's Republic of China csm@fzu.edu.cn

## Abstract

In this study, we synthesized multi-nuclear indium oxide clusters (InOCs) using 1,1′-ferrocene dicarboxylic acid (H_2_FcDCA) as the chelating and surface protection ligand. The obtained clusters include the cubane-type heptanuclear InOCs ([In_7_]) and the sandwich-type thirteen-nuclear InOCs ([In_13_]). Notably, [In_13_] represents the highest nuclear number reported within the InOC family. In addition, the presence of labile coordination sites in these clusters allowed for structural modification and self-assembly. A series of [In_7_] clusters with adjustable band gaps have been obtained and the self-assembly of [In_7_] clusters resulted in the formation of an Fe-doped dimer, [Fe_2_In_12_], and an imidazole-bridged tetramer, [In_28_]. Similarly, in the case of [In_13_] clusters, the coordinated water molecules could be replaced by imidazole, methylimidazole, and even a bridged carboxylic acid, allowing the construction of one-dimensional extended structures. Additionally, part of the H_2_FcDCA could be substituted by pyrazole. This flexibility in replacing solvent molecules offered diverse possibilities for tailoring the properties and structures of the InOCs to suit specific applications.

## Abstract 2

Presented here are a series of ferrocene-functionalized high-nuclear indium-oxo clusters (InOC), containing thirteen-nuclear and cubane-type heptanuclear InOCs. The self-assembly of them leads to the formation of dimers, tetramers, and one-dimensional extended structures.

## Graphical abstract

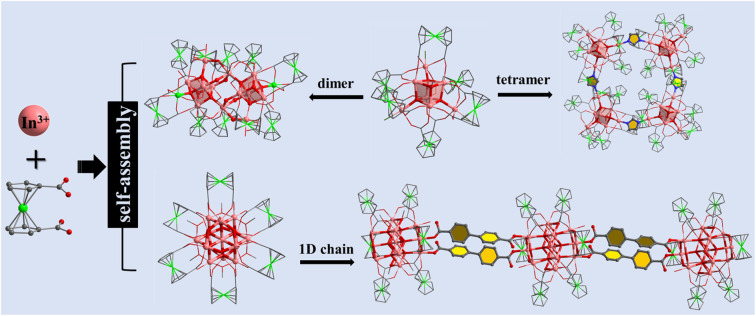

## Introduction

Indium oxide (In_2_O_3_) as an n-type semiconductor possesses excellent electronic and catalytic properties, making it highly promising in various fields.^1–3^ Many methods have been developed to prepare nano-In_2_O_3_,^4^ high-pressure modified In_2_O_3_,^5,6^ and indium-containing mixed oxides and zeolites,^7–9^ due to their application in sensors,^10^ electronics,^11^ and catalysis.^12^ In comparison to In_2_O_3_, indium oxide clusters (InOCs) provide clear structural information and allow for atomic-level control of cluster size. Consequently, they can be used as molecular mimics to explore functional-oriented structural design and optimize the performance of In_2_O_3_ nanomaterials.^13,14^ However, the research on InOCs remains relatively limited, with reported InOCs having low nuclear numbers such as [In_2_],^15–17^, [In_4_],^18^, [In_5_],^19–21^, [In_6_],^22–24^, [In_7_],^25^, [In_10_],^20^ and [In_12_].^14^ In 2006, Neumüller's group synthesized the largest decanuclear InOCs (denoted as [In_10_]) known at that time by utilizing InMe_3_ as the indium source.^20^ Subsequently, the synthesis of high nuclear InOCs has faced stagnation, and their corresponding applications have been scarcely explored. However, the pursuit of crystalline InOCs persisted, driven by the recognition of their promising properties for various technological advancements.

To achieve the synthesis of crystalline InOCs, researchers recognized the crucial importance of slowing down the hydrolysis of In^3+^ ions, as this process greatly influences the formation of well-defined crystal structures. The recently developed coordination delayed hydrolysis (CDH) strategy has shown great potential in the synthesis of crystalline metal-oxo clusters.^26,27^ Inspired by this, we successfully synthesized a series of bixbyite like In_12_-oxo clusters by using diethanol amine as the chelating ligand to control the hydrolysis of In^3+^ ions.^14^ Despite these initial successes, it is crucial to continue delving into the structural diversity and self-assembly behaviors of InOCs (indium-oxo clusters) to unlock their full potential and broaden their applications. However, research in this field remains severely limited.

Compared to diethanolamine, 1,1′-ferrocene dicarboxylic acid (H_2_FcDCA) offers several advantageous features that make it an ideal chelating ligand for synthesizing InOCs:

(a) Versatility in coordination: H_2_FcDCA is widely employed in the construction of coordination compounds due to its flexible conformation and coordination modes.^28–30^ This flexibility allows for various bonding arrangements with metal ions, which can lead to the formation of diverse InOC structures and potentially higher nuclearity.

(b) Remarkable redox and photoelectrocatalytic activity: the ferrocene unit in H_2_FcDCA possesses exceptional redox and photoelectrocatalytic activity, which has garnered significant attention in the field of metal-oxo clusters (such as Sn,^31^ Fe,^32–34^ Co,^35^ Zn,^36^ Mn,^37,38^ Ti^28,29,39–43^). The incorporation of such active units into InOCs could introduce intriguing properties and functionalities to InOCs, making them attractive candidates for various electrochemical and catalytic applications.

(c) Strong coordination ability: the presence of multiple carboxylate groups in H_2_FcDCA facilitates strong coordination with In^3+^ ions. This strong coordination bond effectively protects InOCs and enhances their stability, making them more robust and durable under various conditions.

Due to these advantages, the combination of H_2_FcDCA and InOCs holds the potential to yield high-nuclear InOCs with unique properties and enhanced performance. However, no examples of InOCs functionalized with ferrocene have been reported thus far.

**Scheme 1:**
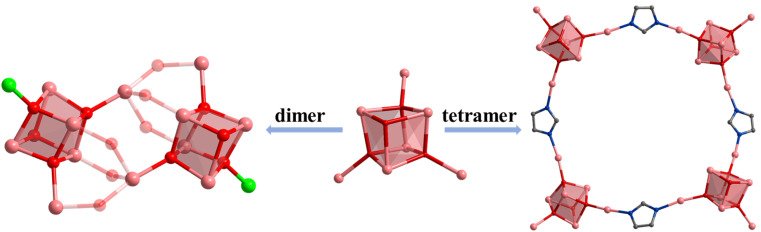
Aggregate assembly of indium oxide clusters. Color: pink, In; red, O; blue, N; gray, C.

Based on the above considerations, H_2_FcDCA was selected as the chelating ligand to react with InCl_3_ or In(NO_3_)_3_, resulting in the synthesis of two distinct groups of InOCs: cubane-type heptanuclear InOCs ([In_7_], compounds 1–4) and sandwich-type thirteen-nuclear InOCs ([In_13_], compounds 5–9) (Table 1). Notably, [In_13_] represents the highest nuclear number recorded within the InOC family. Furthermore, the terminal coordinated solvents in InOCs exhibit lability, making them easily replaceable by other ligands. Consequently, they can serve as secondary building blocks (SBUs) for the formation of dimers, tetramers, and even a one-dimensional extended structure (Scheme 1). Significantly, the inclusion of ferrocene units within these structures endows them with notable redox activity and excellent photocatalytic performance.

**Table 1:** A summary of compounds 1–9^a^

Complex	Composition	Space group	*a* [Å]	*b* [Å]	*c* [Å]	*V* [Å^3^]
1	(TPP^+^)[In_7_FcDCA_6_(μ_4_-O^2−^)_3_(μ_3_-OCH_3_)(Cl^−^)_3_]	*R*3̄	16.1506(10)	16.1506(10)	65.6859(3)	14 838.2(2)
2	(H^+^)_8_[In_12_Fe^II^FcDCA_10_(μ_4_-O^2−^)_6_(μ_3_-O^2−^)_2_(μ_2_-O^2−^)_6_(H_2_O)_6_]	*P*2_1_/*n*	15.4037(2)	20.7536(3)	25.1730(3)	8032.26(18)
3	(H^+^)[In_7_FcDCA_6_(μ_4_-O^2−^)_3_(μ_3_-OCH_3_) (MPP^−^)_3_]	*R*3̄	25.3175(3)	25.3175(3)	29.8382(4)	16 563.2(5)
4	[In_28_FcDCA_24_(μ_4_-O^2−^)_12_(μ_3_-OCH_3_)_4_(Im^−^)_4_(OH^−^)_4_]	*P*4_2_/*n*	35.1846(4)	35.1846(4)	15.8530(4)	19 625.3(7)
5	(H^+^)_3_[In_13_FcDCA_6_(μ_4_-O^2−^)_6_(μ_2_-O^2−^)_6_ (μ_2_-OCH_3_)_6_(H_2_O)_6_]	*C*2/*c*	32.0443(7)	14.9493(3)	28.0793(7)	13 415.8(5)
6	(H^+^)_3_[In_13_FcDCA_6_(μ_4_-O^2−^)_6_(μ_2_-O^2−^)_6_(μ_2_-OCH_3_)_6_(HIm)_6_]	*R*3̄	19.8087(10)	19.8087(10)	28.7268(2)	9761.79(12)
7	(H^+^)_3_[In_13_FcDCA_6_(μ_4_-O^2−^)_6_(μ_2_-O^2−^)_6_(μ_2_-OCH_3_)_6_(DMF)_2_(2-mim)_2_(H_2_O)_2_]	*I*2/*a*	27.37206(19)	15.00755(12)	31.8968(3)	13 070.05(17)
8	(H^+^)_9_[In_13_FcDCA_4_(μ_4_-O^2−^)_6_(μ_2_-O^2−^)_6_(μ_2_-OCH_3_)_6_(OCH_3_)_4_(Cl^−^)_2_(Py^−^)_4_]	*C*2/*m*	19.6313(8)	22.9290(6)	13.3713(4)	5987.4(3)
9	(H^+^)_7_[In_13_FcDCA_6_(μ_4_-O^2−^)_6_(μ_2_-O^2−^)_6_(μ_2_-OCH_3_)_6_(H_2_BPDC)_2_(H_2_O)_2_]	*C*2/*m*	21.2041(3)	25.0454(3)	13.8544(2)	7145.74(18)

a Abbreviations: TPP = tetraphenylphosphine; MPP = *N*-methylpiperazine; HIm = imidazole; DMF = *N*,*N*′-dimethylformamide; 2-mim = 2-methylimidazole; HPy = pyrazol; H_2_BPDC = 4,4′-biphenyldicarboxylic acid.

## Experimental

### Materials and instruments

All the reagents and solvents were purchased commercially and were used as received without further purification. 1,1′-ferrocene dicarboxylic acid (H_2_FcDCA, 99%), InCl_3_ (99%), In(NO_3_)_3_·*x*H_2_O (99%), tetraphenylphosphonium bromide (TPPBr, 99%), imidazole (HIm, 99%), acetic acid (AcOH, 99%), 2-methylimidazole (2-mim, 99%), pyrazol (HPy, 99%), and 4,4′-biphenyldicarboxylic acid (H_2_BPDC, 99%) were acquired from Aladdin Chemical Reagent Shanghai. *N*,*N*′-diethylformamide (DEF, 99%), *N*,*N*′-dimethylformamide (DMF, 99%), triethylamine (99%), methanol (MeOH, 99%), *N*-methylformamide (NMF, 99%), and *N*-methylpiperazine (MPP, 99%) were bought from Sinopharm Chemical Reagent Beijing.

IR spectra (KBr pellets) were recorded on an ABB Bomem MB102 spectrometer over the 400–3900 cm^−1^ range. Powder X-ray diffraction (PXRD) data were collected on a Rigaku Mini Flex II diffractometer using CuKα radiation (*λ* = 1.54056 Å) under ambient conditions. The UV-vis diffuse reflection data were recorded at room temperature using a powder sample with BaSO_4_ as a standard (100% reflectance) on a PerkinElmer Lamda-950 UV spectrophotometer and scanned at 200–1200 nm. Metal proportional analyses were performed on an Ultima-2 inductively coupled plasma (ICP) spectrometer. The TGA curves were recorded in the region of 30–800 °C using a heating rate of 10 °C min^−1^ in a flowing N_2_ atmosphere on a Mettler Toledo TGA/SDTA 851 analyzer.

#### Synthesis of compound 1

A mixture of TPPBr (42.0 mg, 0.10 mmol), H_2_FcDCA (54.0 mg, 0.20 mmol), InCl_3_·4H_2_O (30.0 mg, 0.10 mmol), triethylamine (200 μL, 2.7 mmol), and 4 mL of *N*,*N*′-dimethylformamide (DMF) and methanol (MeOH) (v/v, 1 : 1) was added to 23 mL glass vials respectively, sealed with ultrasound treatment for 5 minutes, and heated in a 100 °C oven for 3 days to generate yellow crystals (yield: 25.0%). FTIR (KBr, cm^−1^): 3340(v), 3236(w), 2352(s), 1659(s), 1582(s), 1540(m), 1476(m), 1387(s), 1360(w), 1293(w), 1190(s), 1031(s), 1106(v), 1036(s), 963(w), 918(s), 868(m), 812(w), 671(s), 868(m), 592(s), 552(s), 493(s), 446(m).

#### Synthesis of compound 2

A mixture of TPPBr (42.0 mg, 0.10 mmol), H_2_FcDCA (27.0 mg, 0.10 mmol), In(NO)_3_·*x*H_2_O (30.0 mg, 0.10 mmol), triethylamine (200 μL, 2.7 mmol), and 4 mL of *N*-methylpiperazine (MPP) and methanol (MeOH) (v/v, 1 : 1) was added to 23 mL glass vials respectively, sealed with ultrasound treatment for 5 minutes, and heated in a 100 °C oven for 5 days and yellow crystals were obtained (yield: 21.0%). FTIR (KBr, cm^−1^): 3140(m), 2939(v), 2355(s), 2323(s), 1612(s), 1594(s), 1483(m), 1395(s), 1289(v), 1197(w), 1143(s), 1017(s), 992(w), 925(w), 876(m), 821(s), 777(m), 617(w), 671(s), 587(s), 516(s), 488(s), 451(m).

#### Synthesis of compound 3

A mixture of TPPBr (42.0 mg, 0.10 mmol), H_2_FcDCA (27.0 mg, 0.10 mmol), In(NO)_3_·*x*H_2_O (30.0 mg, 0.10 mmol), triethylamine (200 μL, 2.7 mmol), and 4 mL of *N*-methylformamide (NMF) and acetonitrile (MeCN) (v/v, 1 : 1) was added to 23 mL glass vials respectively, sealed with ultrasound treatment for 5 minutes, and heated in a 100 °C oven for 5 days and yellow crystals were obtained (yield: 25.0%). FTIR (KBr, cm^−1^): 3388(s), 3090(m), 2935(s), 2838(m), 2368(m), 2335(m), 1640(w), 1558(s), 1483(m), 1475(s), 1464(m), 1382(s), 1370(s), 1338(s), 1024(m), 918(w), 826(w), 769(w), 661(w), 617(w), 568(s), 523(s), 493(s), 420(s).

#### Synthesis of compound 4

A mixture of TPPBr (42.0 mg, 0.10 mmol), H_2_FcDCA (54.0 mg, 0.20 mmol), HIm (7.0 mg, 0.10 mmol), In(NO)_3_·*x*H_2_O (60.0 mg, 0.20 mmol), triethylamine (200 μL, 2.7 mmol), and 4 mL of DMF and MeOH (v/v, 1 : 1) was added to 23 mL glass vials respectively, sealed with ultrasound treatment for 5 minutes, and heated in a 120 °C oven for 7 days and yellow crystals were obtained (yield: 10.0%). FTIR (KBr, cm^−1^): 3291(v), 3100(m), 2933(m), 2839(m), 2397(m), 1676(w), 1584(v), 1478(s), 1387(s), 1355(w), 1251(w), 1197(w), 1083(s), 1027(w), 980(w), 945(s), 918(w), 824(w), 787(w), 668(s), 617(w), 580(s), 521(s), 483(s), 446(s).

#### Synthesis of compound 5

A mixture of TPPBr (42.0 mg, 0.10 mmol), H_2_FcDCA (14.0 mg, 0.20 mmol), acetic acid (AcOH, 30 μL, 0.47 mmol), In(NO)_3_·*x*H_2_O (30.0 mg, 0.10 mmol), triethylamine (200 μL, 2.7 mmol), and 4 mL of DMF and MeOH (v/v, 1 : 1) was added to 23 mL glass vials respectively, sealed with ultrasound treatment for 5 minutes, and heated in a 100 °C oven for 2 days and yellow crystals were obtained (yield: 28.0%). FTIR (KBr, cm^−1^): 3250(m), 2929(m), 2827(m), 1664(s), 1555(s), 1491(m), 1464(m), 1390(w), 1370(w), 1190(w), 1007(w), 925(w), 826(w), 785(s), 573(s), 524(s), 476(w), 422(s).

#### Synthesis of compound 6

A mixture of TPPBr (42.0 mg, 0.10 mmol), H_2_FcDCA (14.0 mg, 0.20 mmol), HIm (7.0 mg, 0.10 mmol), In(NO)_3_·*x*H_2_O (120.0 mg, 0.40 mmol), triethylamine (200 μL, 2.7 mmol), and 4 mL of *N*,*N*′-diethylformamide (DEF) and MeOH (v/v, 1 : 1) was added to 23 mL glass vials respectively, sealed with ultrasound treatment for 5 minutes, and heated in a 100 °C oven for 6 days and yellow crystals were obtained (yield: 27.0%). FTIR (KBr, cm^−1^): 3139(m), 2935(m), 2828(m), 2368(m), 2335(m), 1651(s), 1560(m), 1491(m), 1390(m), 1370(m), 1325(w), 1258(w), 1202(w), 1103(s), 1079(s), 1017(v), 943(s), 923(w), 864(s), 775(m), 649(w), 612(w), 518(s), 419(s).

#### Synthesis of compound 7

A mixture of TPPBr (42.0 mg, 0.10 mmol), H_2_FcDCA (14.0 mg, 0.20 mmol), 2-methylimidazole (8.0 mg, 0.10 mmol), In(NO)_3_·*x*H_2_O (120.0 mg, 0.40 mmol), AcOH (30 μL, 0.47 mmol), triethylamine (200 μL, 2.7 mmol), and 4 mL of DMF and MeOH (v/v, 1 : 1) was added to 23 mL glass vials respectively, sealed with ultrasound treatment for 5 minutes, and heated in a 100 °C oven for 2 days and yellow crystals were obtained (yield: 25.0%). FTIR (KBr, cm^−1^): 3080(w), 2927(m), 2827(s), 2365(s), 2335(m), 1654(w), 1560(s), 1483(m), 1456(m), 1392(w), 1365(s), 1190(s), 1090(s), 1017(s), 913(w), 824(w), 777(w), 654(w), 580(s), 466(v), 414(s).

#### Synthesis of compound 8

A mixture of TPPBr (42.0 mg, 0.10 mmol), H_2_FcDCA (14.0 mg, 0.20 mmol), In(NO)_3_·*x*H_2_O (120.0 mg, 0.40 mmol), pyrazol (HPy, 6.8 mg, 0.10 mmol), triethylamine (200 μL, 2.7 mmol), and 4 mL of DEF and MeOH (v/v, 1 : 1) was added to 23 mL glass vials respectively, sealed with ultrasound treatment for 5 minutes, and heated in a 100 °C oven for 6 days to generate several yellow plate crystals of 8 and a large amount of unidentified precipitate. Only their crystal structures are described below. (Yield: 26.0%). FTIR (KBr, cm^−1^): 3280(m), 3095(m), 2975(s), 2930(m), 2825(s), 2335(m), 1646(w), 1548(v), 1496(v), 1392(w), 1372(w), 1300(v), 1202(w), 1100(s), 1014(s), 920(w), 842(w), 780(s), 580(s), 516(s), 474(s), 424(v).

#### Synthesis of compound 9

A mixture of TPPBr (42.0 mg, 0.10 mmol), 4,4′-biphenyldicarboxylic acid (H_2_BPDC, 12.0 mg, 0.05 mmol), H_2_FcDCA (14.0 mg, 0.05 mmol), In(NO)_3_·*x*H_2_O (60.0 mg, 0.20 mmol), triethylamine (200 μL, 2.7 mmol), and 5 mL of formamide and polyethylene glycol (v/v, 1 : 1) was added to 23 mL glass vials respectively, sealed with ultrasound treatment for 5 minutes, and heated in a 100 °C oven for 14 days and yellow crystals were obtained (yield: 11.0%). FTIR (KBr, cm^−1^): 3480(v), 3079 (m), 2930(m), 2830(s), 1654(m), 1572(s), 1491(s), 1387(m), 1353(m), 1187(s), 1091(s), 1010(s), 925(s), 834(s), 757(v), 666(m), 582(w), 508(s), 471(s), 424(m).

### X-Ray crystallographic analysis

Single crystal X-ray diffraction data of porous materials were collected using a Hybrid Pixel Array detector equipped with Ga Kα radiation (*λ* = 1.3405 Å) at about 298 K and 100 K. The structures were solved with the dual-direct methods using ShelXT and refined with the full-matrix least-squares technique based on F_2_ using SHELXL. Non-hydrogen atoms were refined anisotropically. Hydrogen atoms were added theoretically, riding on the concerned atoms and refined with fixed thermal factors. All absorption corrections were performed using the multi-scan program.

### Photocatalytic measurements

A 25 mL tube was charged with 10 mg of sample powder, 10 mg [Ru(bpy)_3_]Cl_2_·6H_2_O, 8 mL MeCN, 2 mL H_2_O and 32.1 mg 1,3-dimethyl-2-phenylbenzimidazoline (BIH). It was ultrasonicated for 20 min to obtain a well-dispersed suspension. Then the resulting suspension was transferred into a Pyrex side-irradiation reaction vessel connected to a closed gas system. The reaction mixture was irradiated by visible light generated by a 300 W Xe light-source (PerfectLight, PLS-SXE300/300UV) with a 420 nm cut-off filter. The generated gas products were analyzed by a gas chromatography analyzer (FULI 9790II) equipped with a flame ionization detector (FID) and thermal conductivity detector (TCD). The product evolution rates were determined from a linear regression fit.

## Results and discussion

**Fig. 1:**
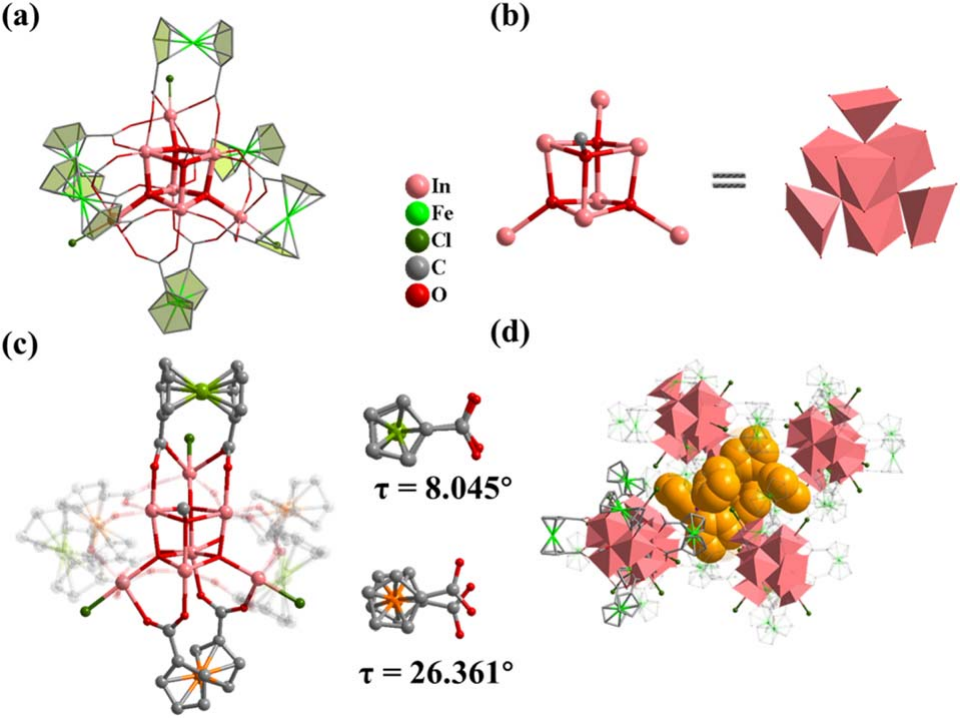
(a) Structure of 1; (b) the cubane central ion is [In_7_(μ_4_-O^2−^)_3_(μ_3_-OCH_3_)]^14+^; (c) coordination environment of 1; (d) 1 accumulates along the *c*-axis and is filled with TPP^+^. Color: pink, In; green, Fe; red, O; blue, N; gray, C; dark green, Cl; yellow, TPP^+^.

Compound 1 was obtained by using InCl_3_·4H_2_O as the metal source in DMF and MeOH mixed solvents. Single crystal X-ray diffraction analysis shows that 1 crystallizes in the hexagonal *R*3̄ space group with the formula TPP^+^[In_7_FcDCA_6_(μ_4_-O^2−^)_3_(μ_3_-OCH_3_)(Cl^−^)_3_]^−^ (TPPBr = tetraphenylphosphonium bromide), which contains an anionic cluster [In_7_FcDCA_6_(μ_4_-O^2−^)_3_(μ_3_-OCH_3_)(Cl^−^)_3_]^−^ and a free cationic guest, TPP^+^. Its cluster nucleus contains a twisted cubane central ion [In_7_(μ_4_-O^2−^)_3_(μ_3_-OCH_3_)]^14+^ which was stabilized by six FcDCA^2−^ ligands accompanied by three chlorides (Fig. 1a). In 1, the In^3+^ ion is six coordinated, and four In^3+^ ions are linked by three μ_4_-O^2−^ and a μ_3_-OCH_3_ to form a twisted hexahedron (Fig. 1b). The In–O bond distance ranges from 2.120 Å to 2.384 Å (Fig. S1†), which were comparable with those reported in the literature.^44,45^1 is different from the previously reported [Fe_7_(μ_4_-O)_3_(μ_3_-OCH_3_)]^14+^.^32^ The cluster nuclei are isomorphic. The periphery of the cluster nucleus of 1 is modified by three terminal Cl^−^ ions and six FcDCA^2−^ ligands. The FcDCA^2−^ ligand is divided into two groups: one group in which the carboxyl torsion angle of FcDCA^2−^ ligands is relatively small (8.045°) and which links three In(iii) ions in η^1^:η^1^:η^1^:η^1^:μ_3_ mode, while the other group (26.361°) links four In(iii) ions in η^1^:η^1^:η^1^:η^1^:μ_4_ mode (Fig. 1c). Due to the existence of TPP^+^, the stacking of 1 along the *c*-axis is relatively dense (Fig. 1d).

**Fig. 2:**
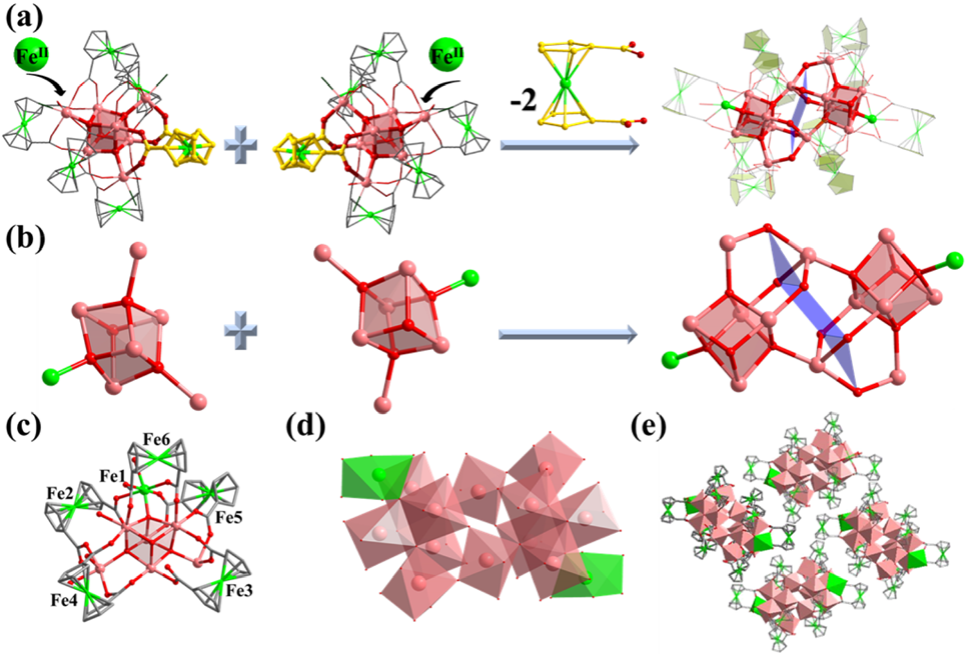
Molecular structure of compound 2. (a) Assembly of the In atom in 2; (b) two cubane central ions [In_6_Fe(μ_4_-O^2−^)_3_(μ_3_-O^2−^)]^13+^; (c and d) coordination environment in 2 ; (e) packing structure of 2.

Compound 2 was synthesized in NMF (*N*-methylformamide) and MeCN (acetonitrile) mixed solvents. Compound 2 contains an anionic cluster, [In_12_Fe^II^_2_FcDCA_10_(μ_4_-O^2−^)_6_(μ_3_-O^2−^)_2_(μ_2_-O^2−^)_6_(H_2_O)_6_]^8−^. Its cluster core contains an [In_12_Fe^II^_2_(μ_4_-O^2−^)_6_(μ_3_-O^2−^)_2_(μ_2_-O^2−^)_6_]^12+^ coordinated by ten FcDCA^2−^ ligands and six water molecules. 2 can be seen as a dimer formed by two [In_7_] cores of 1. The two outermost In metal centers are replaced by Fe ions which are released by the decomposition of FcDCA^2−^ ligands (Fig. 2a). These structural features and elemental substitutions have been supported by single crystal X-ray diffraction (XRD) and inductively coupled plasma (ICP) analysis results. The two Fe atoms are in the +2 oxidation state, which is speculated by the bond valence sum calculation with a BVS value of *ca.* 2.0 (Tables S3 and S4†).

[In_12_Fe^II^_2_(μ_4_-O^2−^)_6_(μ_3_-O^2−^)_2_(μ_2_-O^2−^)_6_]^12+^ in 2 contains two cubane central ions [In_6_Fe^II^(μ_4_-O^2−^)_3_(μ_3_-O^2−^)]^12+^ and six μ_2_-O^2−^, where four In^3+^ ions are linked by three μ_4_-O^2−^ and one μ_3_-O^2−^ to form a twisted hexahedron (Fig. 2b–d). The stacking of 2 along the *a*-axis is relatively loose (Fig. 2e).

**Fig. 3:**
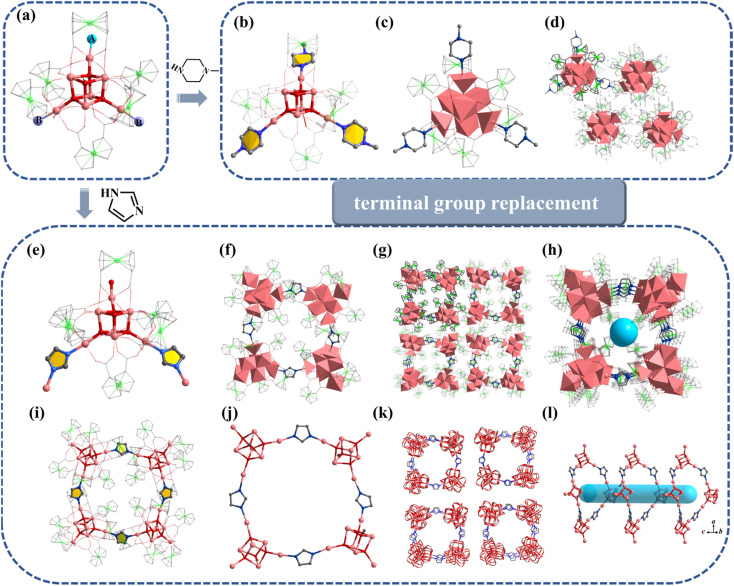
Molecular structure of compound 3. (a) A and B represent substitution sites. (b and c) 3 with MPP ligand. (d) Packing of 3 along the *c*-axis. (e and f) Molecular structure of 4. (g and h) Packing structure of 4. (i–l) Cluster assembly in 4. The atom color code: pink, In; green, Fe; red, O; blue, N; gray, C; dark green, Cl.

Compound 3 was obtained by introducing 1-methylpiperazine (HMPP) in the reaction system of 1, wherein the chlorine terminal sites of 1 (Fig. 3a, site A and site B) are replaced by MPP^−^. Compound 3 also crystallizes in hexagonal *R*3̄ space group with containing an anionic cluster [In_7_FcDCA_6_(μ_4_-O^2−^)_3_(μ_3_-OCH_3_)(MPP^−^)_3_]^−^, which contains a cubane central ion [In_7_(μ_4_-O^2−^)_3_(μ_3_-OCH_3_)]^14+^ and six FcDCA^2−^ ligands and three MPP^−^ ligands (Fig. 3b and c). The stacking of 3 is relatively loose (Fig. 3d).

Furthermore, the [In_7_] core of 1 can be used as SBU to construct a molecular ring. Compound 4 was obtained by adding imidazole (HIm) in the reaction system of 1, resulting in the replacement of site A by an Im^−^ bridged ligand to form a tetramer, [In_28_] ring (Fig. 3e–l). Compound 4 crystallizes in tetragonal *P*4_2_/*n* space group and contains four twisted cubane central ions [In_7_(μ_4_-O^2−^)_3_(μ_3_-OCH_3_)]^14+^, twenty-eight FcDCA^2−^ ligands, four Im^−^ ions and four OH^−^ ions (Fig. 3e and f). The coordination environment of [In_7_(μ_4_-O^2−^)_3_(μ_3_-OCH_3_)]^14+^ is similar to 1, except that the two terminal chlorine atom sites are replaced by the Im-ligand (Fig. 3f, i and j). The In–O bond distance ranges from 2.099 Å to 2.290 Å. The In–N bond distance ranges from 2.146 Å to 2.171 Å. Therefore, 4 can be seen as the tetramer of 1. The center of the ring can be stacked along the *c*-axis to form 1D channels (Fig. 3g, h, k and l). It is worth mentioning that this [In_28_] ring holds the largest size record among InOCs (Fig. S2 and S9†).

Inspired by the tremendous success of using monodentate carboxylic acid as a regulator in constructing high-nuclear metal–oxygen clusters and high-valence metal–organic frameworks (MOFs),^46–53^ acetic acid was further introduced into the synthesis system, and a series of 13 nuclear In_13_-oxo clusters were successfully synthesized. These In_13_-oxo clusters represent the highest nuclear number in InOCs.

**Fig. 4:**
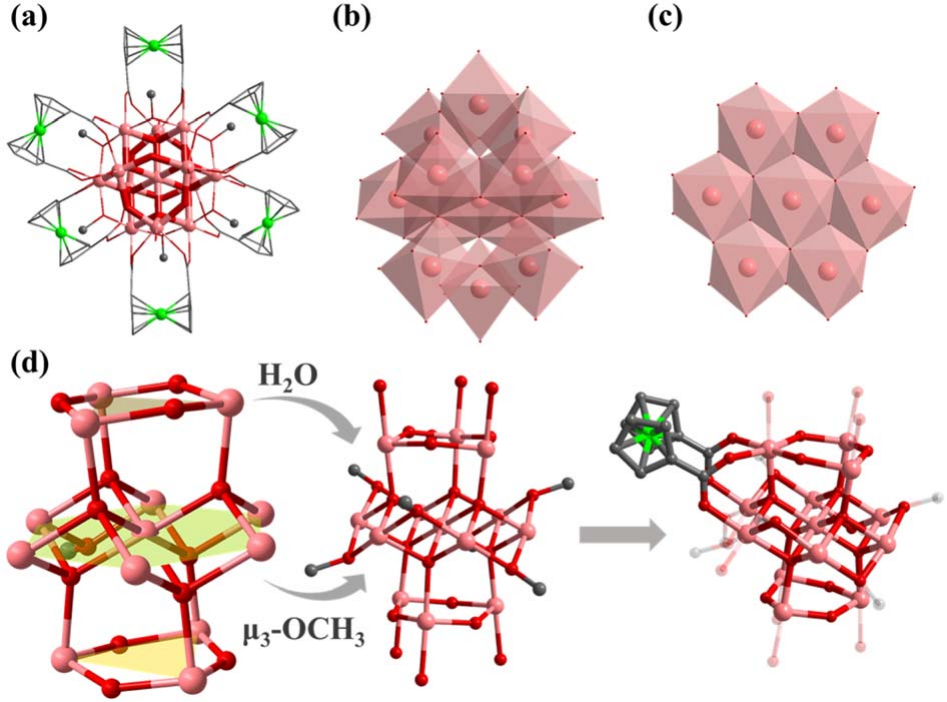
Molecular structure of 5: (a) structure of 5; (b) the cubane central ion is [In_13_(μ_4_-O^2−^)_6_(μ_2_-O^2−^)_6_]^15+^; (c) [In_7_(μ_4_-O^2−^)_6_(μ_3_-OCH_3_)_6_]^3+^ is the Anderson configuration; (d) coordination environment of [In_13_(μ_4_-O^2−^)_6_(μ_2_-O^2−^)_6_]^15+^. Color: pink, In; green, Fe; red, O; blue, N; gray, C; dark green, Cl.

Compound 5 crystallizes in monoclinic *C*2/*c* space group and contains an [In_13_(μ_4_-O^2−^)_6_(μ_2_-O^2−^)_6_]^15+^ core, which was stabilized by six FcDCA^2−^ ligands accompanied by six deprotonated methanol molecules and six water molecules. [In_13_(μ_4_-O^2−^)_6_(μ_2_-O^2−^)_6_]^15+^ can be viewed as a sandwich-type trilayer structure (Fig. 4a–d). The middle part is an Anderson-type [In_7_(μ_4_-O^2−^)_6_]^9+^ [In_7_],^54^ which is a hexagonal planar ring formed by seven coplanar In(iii) ions through six μ_4_-O^2−^ bridges. Each μ_4_-O^2−^ is connected to three In(iii) ions, forming a coplanar tetrahedron. And the upper and lower parts are approximately equilateral triangle [In_3_(μ_2_-O^2−^)_3_]^6+^. The upper and lower layers of [In_3_] form two equilateral triangles with a side length of 3.85 Å, and the angle of In–O–In is 136.10°. A similar cluster core has been reported in the cobalt(ii)-containing arsenomolybdate [Co(H_2_O)_6_]K_2_[As_6_CoMo_6_O_30_],^55^ but it is the first discovery in the field of indium oxide clusters. Six methanol molecules are uniformly connected around the planar ring of [In_13_(μ_4_-O^2−^)_6_(μ_2_-O^2−^)_6_]^15+^ to form an [In_13_(μ_4_-O^2−^)_6_(μ_2_-O^2−^)_6_(μ_3_-OCH_3_)_6_]^9+^. The outermost edge of the anionic cluster [In_13_] is modified by six FcDCA^2−^ ligands, with two carboxyl groups on each FcDCA^2−^ ligand in η^1^:η^1^:η^1^:η^1^:μ_3_ modes bridging three In atoms to form [In_13_FcDCA_6_(μ_4_-O^2−^)_6_(μ_2_-O^2−^)_6_(μ_3_-OCH_3_)_6_]^3−^ (Fig. 4d). It is similar to the layer previously reported [Mn_13_].^38^ It is different from the cluster core configuration of [In_13_], which may be due to the different coordination modes of FcDCA^2−^ ligands.

**Fig. 5:**
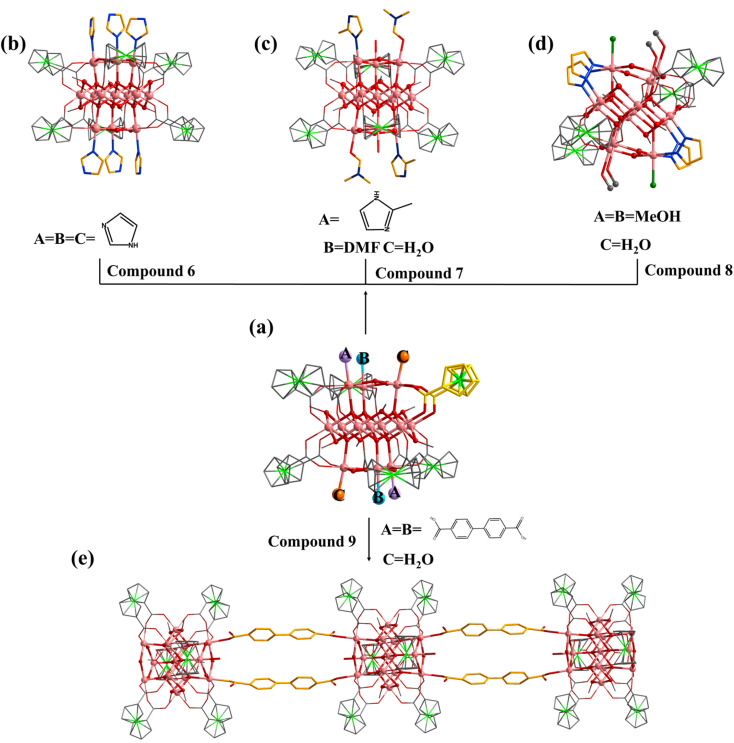
(a) [In_13_] as a secondary building unit: (b) compound 6 (A = B = C = HIm); (c) compound 7 (A= 2-mim, B = DMF, C = H_2_O); (d) compound 8 (A = B = MeOH, C = H_2_O); (e) compound 9 (A = B = BPDC, C = H_2_O. A, B and C represent different substitution sites).

Similar to 1, the terminal coordination sites of [In_13_] (Fig. 5a, site A, site B and site C) can be replaced by diverse molecules. All sites (A, B and C) are replaced by HIm to obtain compound 6 with the formula [In_13_FcDCA_6_(μ_4_-O^2−^)_6_(μ_2_-O^2−^)_6_(μ_2_-OCH_3_)_6_(HIm)_6_]^3−^ (Fig. 5b). Compound 7 was obtained by using 2-methylimidazole (2-mimH) to replace HIm (Fig. 5c).

Compound 7 contains an anionic [In_13_FcDCA_6_(μ_4_-O^2−^)_6_(μ_2_-O^2−^)_6_(μ_2_-OCH_3_)_6_]^3−^ core, and the sites A, B and C are occupied by 2-mim, DMF, and H_2_O respectively.

Pyrazole (HPy) was added to replace HIm resulting in the formation of compound 8 (Fig. 5d). Compound 8 contains an anionic cluster [In_13_FcDCA_4_(μ_4_-O^2−^)_6_(μ_2_-O^2−^)_6_(μ_2_-OCH_3_)_6_(OCH_3_)_4_Cl^−^_2_(Py^−^)_4_]^9−^. Compared to 5, sites A and B are replaced by methanol molecules in 8. Additionally, due to the similarity of the Py^−^ coordination mode to the carboxyl group, two FcDCA^2−^ ligands are replaced by four chelating Py^−^ ligands.

Interestingly, 4,4′-biphenyldicarboxylic acid (H_2_BPDC) was utilized as a bridged ligand to construct a one-dimensional extended structure based on [In_13_], compound 9. Compound 9 contains an anionic cluster [In_13_FcDCA_6_(μ_4_-O^2−^)_6_(μ_2_-O^2−^)_6_(μ_2_-OCH_3_)_6_(BPDC)_2_(H_2_O)_2_]^7−^ similar to that of 5 (Fig. 5e). Sites A and B are replaced by 4,4′-biphenyldicarboxylic acid in 9 to form a chain along the *a*-axis. The description of structural details of compounds 5–9 is provided in the ESI† (Fig. S10–S12). Physical characterizations such as powder X-ray diffraction (PXRD), infrared spectroscopy and thermogravimetry analyses revealed the purity and composition of the samples from a macroscopic perspective, further corroborating the single-crystal characterization results (Fig. S13–S30†).

Here, we summarize the synthesis and structure:

(1) By introducing monodentate carboxylic acid as a regulator, we achieve a metal cluster core design from low to high nuclei;

(2) by introducing auxiliary ligands to obtain dimers, tetramers, and even one-dimensional extended structures, the end coordination solvents of [In_7_] and [In_13_] exhibit instability, serving as SBUs.

### Electrochemical measurements

**Fig. 6:**
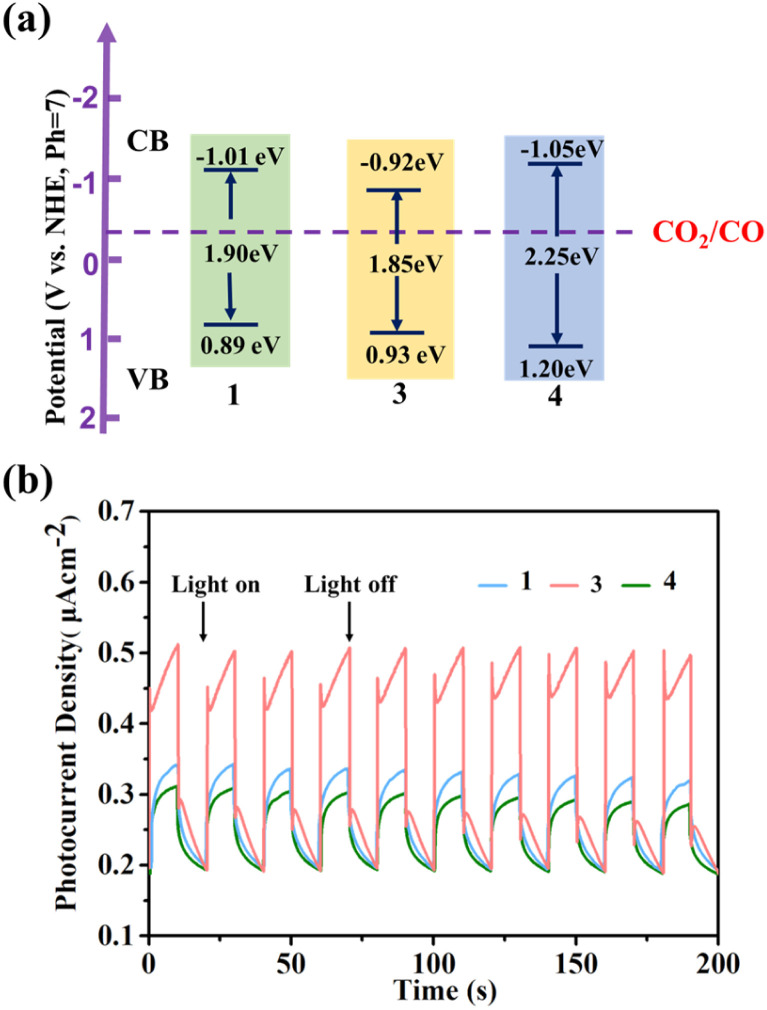
(a) Energy-level diagram of 1, 3 and 4; (b) 0.4 V-bias photocurrent responses of electrodes derived for 1, 3 and 4 in 0.2 M Na_2_SO_4_ aqueous solution under repetitive chopped visible light irradiation.

Due to its good acid–base stability and thermal stability, 1 has the potential to be used as a photocatalyst material. We investigated the optical properties related to photocatalysis of compounds 1, 3, and 4 with similar structures. The UV absorption spectrum obtained from the solid UV diffuse reflection spectrum conversion shows a very obvious strong absorption peak at around 600 nm in the UV band (Fig. S31–S39†). After the Kubelka Munk equation transformation,^56^ we obtained optical bandgaps of 1.90 eV, 1.85 eV, and 2.25 eV for 1, 3, and 4, respectively (Fig. 6a). We conducted Mott Schottky testing^57^ (Fig. S40–S42†), and the results show that the test curves of 1, 3, and 4 have a positive slope and are n-type semiconductors. By testing at different frequencies, we get the corresponding lowest unoccupied molecular orbital (LUMO) energies of −1.01 eV, −0.92 eV, and −1.05 eV (V *vs.* NHE, pH = 7). Therefore, it is calculated that the highest occupied molecular orbital (HOMO) is 0.89 eV, 0.93 eV and 1.20 eV respectively. From the LUMO energy levels, the LUMO positions of 1, 3, and 4 meet the thermodynamic requirements for carbon dioxide reduction.

Their photocurrent response was studied under visible light irradiation. The experiment was conducted at room temperature in 0.2 M Na_2_SO_4_ electrolyte using a 300 W high-pressure xenon lamp (*λ* > 420 nm) as a visible light source. To eliminate errors caused by uneven film thickness, a back lighting method is used. During the testing period, the xenon lamp is manually shielded to maintain its on–off cycle illumination (with an interval of 10 seconds), and maintain the voltage at 0.4 V. Visible photocurrent directions were observed for compounds 1, 3, and 4. On the one hand, photocurrent is rapidly generated and remains stable, and the intensity has not significantly decreased, indicating that they have good photoelectric response and stability. On the other hand, we found that the response effect showed the following trend: 3 > 1 > 4, which was consistent with the size trend of the UV band gap (Fig. 6b).

### Photocatalytic CO_2_ reduction

**Fig. 7:**
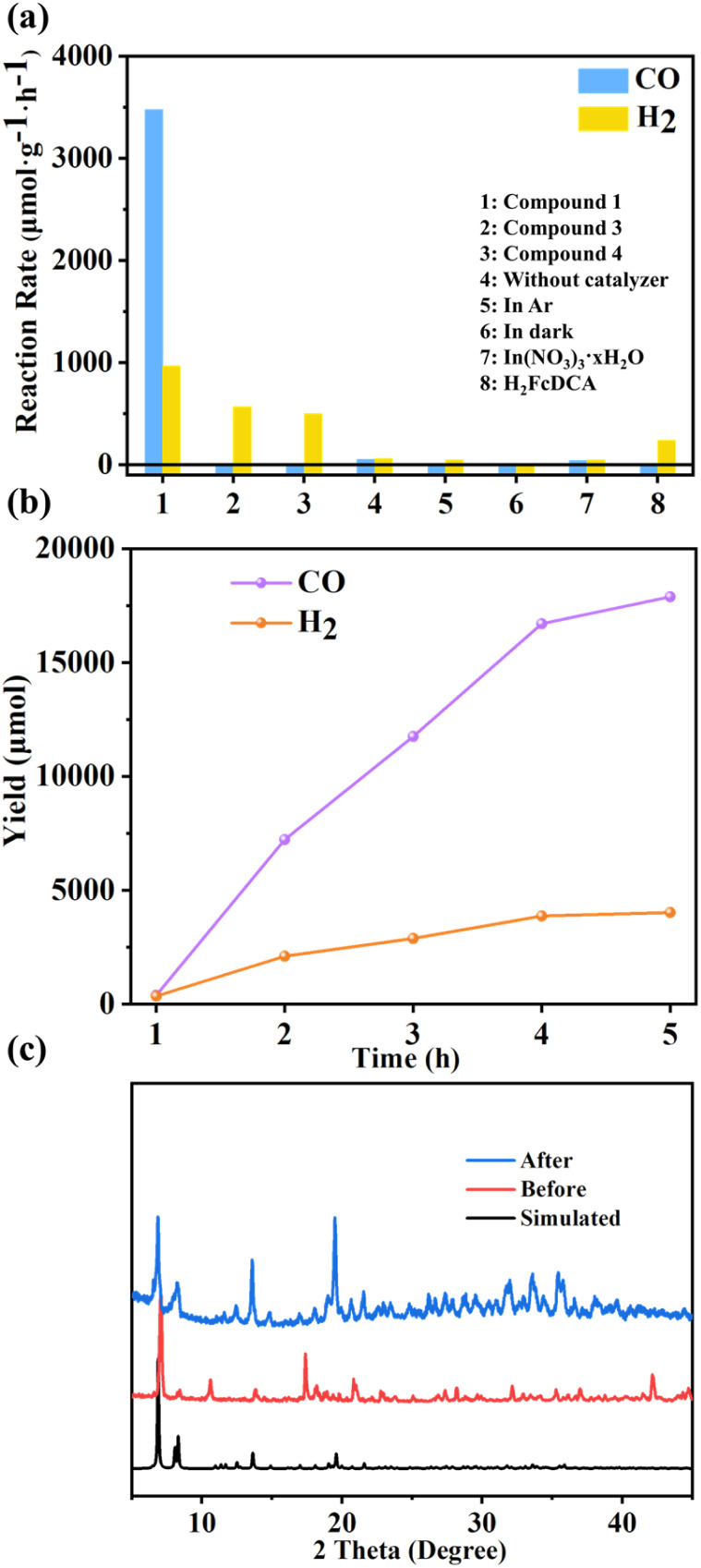
(a) Reaction rates under different conditions; (b) photocatalytic performance test chart of compound 1; (c) powder XRD patterns for compound 1 before and after photocatalytic reaction.

When compounds 1, 3 and 4 were used directly as catalysts for photocatalytic carbon dioxide reduction, discernible photoreduction products were not observed. Consequently, we opted to employ [Ru(bpy)_3_]Cl_2_·6H_2_O as the photosensitizer for the system. Additionally, we utilized 1,3-dimethyl-2-phenylbenzimidazoline (BIH) as the electron sacrificial agent for the reaction. Visible light with wavelengths greater than 420 nm was chosen as the light source with a measured light intensity density of 458 mW cm^2^ using a light intensity meter for the photocatalytic activity testing. Gas chromatography was employed to monitor the gas phase products. It can be seen that after 5 hours of illumination, the systems employing catalysts 1, 3, and 4 respectively achieved CO generation rates of 3477 μmol g^−1^ h^−1^, 11 μmol g^−1^ h^−1^, and 10 μmol g^−1^ h^−1^. Correspondingly, the rates of H_2_ reached 967 μmol g^−1^ h^−1^, 571 μmol g^−1^ h^−1^, and 500 μmol g^−1^ h^−1^ (Fig. 7a and b). The selectivity of 1 pair of CO products reached 82%.

A series of control experiments were also conducted to elucidate the roles of various components within the photocatalytic system. The lack of photosensitizers has led to a significant reduction in products, highlighting the role of photosensitizers in helping catalysts improve light utilization efficiency in the reaction (Fig. S43†). In instances where our cluster was not added, marginal amounts of CO and H_2_ were detected, likely attributed to the inherent photocatalytic activity of the photosensitizer alone. Substituting CO_2_ with Ar yielded negligible carbon product generation, confirming the origin of CO from CO_2_ reduction. In the absence of light, no gas-phase products were detected, confirming the photocatalytic nature of the reaction. Similarly, when BIH was omitted, product formation was not observed, highlighting the essential nature of electron sacrificial agents. Further investigations encompassed the photocatalytic activity of metal salts and ligand H_2_FcDCA. Metal salts exhibited similarity to the scenario involving solely photosensitizers without catalysts, suggesting the absence of significant catalytic activity in metal salts. The system incorporating ligand H_2_FcDCA yielded a hydrogen generation rate of 239.1 μmol g^−1^ h^−1^, along with an average CO production rate of 0.8 μmol g^−1^ h^−1^. This indicates the marginal catalytic impact of H_2_FcDCA in photocatalytic CO_2_ reduction.

Following the reaction, compounds 1, 3, and 4 were recovered through filtration, washing, and drying processes, and their structures were characterized. Post-reaction PXRD data indicated a loss of crystalline states in 3 and 4, evidenced by the broadening and disappearance of peak patterns. In contrast, the PXRD pattern of 1 remained nearly unchanged (Fig. 7c), signifying its stability after the reaction. Compared to the other two materials, the pronounced photocatalytic CO_2_ effect of 1 may be attributed to its superior stability. Notably, 3 and 4 displayed a propensity for hydrogen gas production akin to the catalytic outcomes of H_2_FcDCA. The selectivity of compounds 3 and 4 (pair of H_2_ products) increases, which is probably attributed to the decomposition of compounds 3 and 4 to produce FcDCA^2−^ ligands, mainly for photocatalytic hydrogen production. This trend could potentially stem from the instability of the In–N bond within 3 and 4, leading to decomposition within the catalytic system and resulting in FcDCA^2−^ formation.

On the basis of the above discussion, a feasible mechanism of photocatalytic CO_2_RR can be explained as follows. In^3+^ with d^10^ structure and H_2_FcDCA are extraordinary light-trapping elements.^58–60^ First, many electron–hole pairs are generated in compounds 1, 3, and 4 driven by visible light, and the In^3+^ ions of InOCs can simultaneously obtain the photoexcited electrons migrated from the FcDCA^2−^ ligands and photosensitizers to become reduced indium ions.^58,61^ At the same time, the BIH molecules behave as sacrificial agents to quench the remaining photogenerated holes.^62^ At last, the accepted photoexcited electrons in reduced indium ions further move to the absorbed CO_2_ molecules for the CO_2_ reduction reaction, while reduced indium ions are oxidized to the original In^3+^ ions. Due to the lack of significant catalytic activity in metal salts, the ligand H_2_FcDCA generates H_2_ and trace amounts of CO. That is a good reflection for the effectiveness of our initial design for InOCs as a visible light catalyst by selecting an In(iii) center with d^10^ properties and H_2_FcDCA ligand with strong light absorption.

## Conclusions

In this study, we successfully synthesized InOCs using 1,1′-ferrocene dicarboxylic acid (H_2_FcDCA) as the chelating ligand and surface protection ligand. The cubane-type heptanuclear InOCs ([In_7_]) and sandwich-type thirteen-nuclear InOCs ([In_13_]) were obtained for the first time. Notably, [In_13_] represents the highest nuclear number. The self-assembly of these InOCs results in the formation of a series of dimers, tetramers, and one-dimensional extended structure. The inclusion of ferrocene units within these clusters resulted in remarkable redox activity and exceptional photocatalytic performance. These findings highlight the potential of H_2_FcDCA as a versatile chelating ligand for synthesizing InOCs with enhanced properties. The ability to control the size and structure of InOCs opens up new avenues for their utilization in various applications such as catalysis, optoelectronics, and nanotechnology. Further exploration and application of InOCs with structural diversity are necessary to uncover their full potential and promote their broad range of applications.

## Data availability

Data will be available on request.

## Author contributions

All authors contributed extensively to the work presented in this paper. F. Wang, J. Zhang and S.-M. Chen conceived the research project. R. Zhang performed the synthesis and characterizations and catalytic experiments. J.-J. Lan assisted with the data collection. F. Wang and R. Zhang wrote the manuscript and ESI with input from the other authors.

## Conflicts of interest

There are no conflicts to declare.

## Supplementary Material

SC-015-D3SC05824G-s001

SC-015-D3SC05824G-s002
