# Supplementary material for: Aggregate assembly of ferrocene functionalized indium-oxo clusters
Source: Chem Sci. 2023 Dec 4;15(2):726–35. doi: 10.1039/d3sc05824g (PMC10762979; doi:10.1039/d3sc05824g)
Supplement: SC-015-D3SC05824G-s001 [file SC-015-D3SC05824G-s001.pdf]

Electronic Supplementary Material (ESI) for Chemical Science.
This journal is © The Royal Society of Chemistry 2023

Aggregate Assembly of Ferrocene Functionalized Indium-oxo Clusters for Photocatalysis

Rong Zhang[a, b], Jiajing Lan[a,b] , Fei Wang,*[a] Shu-Mei Chen*[b], Jian Zhang[a*]

[a] State Key Laboratory of Structural Chemistry, Fujian Institute of Research on the Structure of Matter, Chinese Academy of Sciences, Fuzhou, Fujian 350002, P. R. China.

[b] College of Chemistry, Fuzhou University, 350108 Fuzhou, P.R. China.
*E-mails of corresponding authors: wangfei04@fjirsm.ac.cn; zhj@fjirsm.ac.cn; csm@fzu.edu.cn

Contents

1. Electrochemical measurement 2
2. Photocatalytic CO_2 reduction 2
3. Single crystal synthesis and characterization of compounds . 2
    Single crystal structure determination 3
4. Molecular Structure 4
5. Characterizations 8
    PXRD analyses 8
    pH stability and solvent stability 8
    Infrared spectrum analysis 12
    ICP analyses 12
    TGA 13
    UV-Vis spectra 15

1. Electrochemical measurement

We prepared the working electrode by solution coating method as follows: the newly prepared sample (5 mg) and Nafion (10 μL) dissolved in 0.5 mL ethanol with ultrasound and 40 μL solution was uniformly dropped on clean FTO conductive glass (1.0 × 4.0 cm^2, 10 $\Omega \cdot cm^{-2}$). The photocurrent experiment was carried out on the three electrode system of the CHI760E electrochemical workstation, in which Pt sheet was the counter electrode and Ag/AgCl electrode was the reference electrode. The experiment was carried out in 0.2 M Na_2SO_4 electrolyte at room temperature, and a 300 W Xe light-source (PerfectLight, PLS-SXE300/300UV) with a 420 nm cut-off filter was used as a visible light source. Mott-Schottky experiment was carried out on a three electrode system of the electrochemical workstation (IM6, ZAHNER) at frequencies of 500 Hz, 1000 Hz, and 1500 Hz, with a voltage range of -1.0 V to 1.0 V ($V_{vs.}$ NHE, pH=7).

2. Single crystal synthesis and characterization of compounds

Single crystal structure determination

Table S 1. Crystal data and strutures refinement for **1**-**3**.

**Identification code**	**InOC-1**	**InOC-2**	**InOC-3**
**Empirical formula**	$C_{95}H_{66}Cl_3Fe_6In_7O_{28}P$	$C_{120}Fe_{12}In_{12}O_{60}H_{84}$	$C_{88}H_{84}Fe_6In_7N_6O_{28}$
**Formula weight**	2931.63	4533.91	2812.45
**Temperature/K**	293(2)	100.00(10)	100.01(10)
**Crystal system**	hexagonal	monoclinic	hexagonal
**Space group**	*R*-3	$P2_1/n$	*R*-3
**a/Å**	16.15060(10)	15.4037(2)	25.3175(3)
**b/Å**	16.15060(10)	20.7536(3)	25.3175(3)
**c/Å**	65.6859(3)	25.1730(3)	29.8382(4)
**α/°**	90	90	90
**β/°**	90	93.5110(10)	90
**γ/°**	120	90	120
**Volume/Å³**	14838.2(2)	8032.26(18)	16563.2(5)
**Z**	6	2	6
**ρ_{calc}g/cm³**	1.968	1.875	1.692
**Goodness-of-fit on F^2**	1.062	2.714	1.044
**Final R indexes [I>=2σ (I)]**	R_1 = 0.0241, wR_2 = 0.0693	R_1 = 0.2120, wR_2 = 0.5627	R_1 = 0.0450, wR_2 = 0.1279

**Final R indexes [all data]**	R_1 = 0.0259, wR_2 = 0.0701	R_1 = 0.2289, wR_2 = 0.5776	R_1 = 0.0472, wR_2 = 0.1297
**CCDC No.**	2281562	2281565	2281564

Table S 2. Crystal data and strutures refinement for **4-6**.

**Identification code**	**InOC-4**	**InOC-5**	**InOC-6**
**Empirical formula**	$C_{304}H_{216}Fe_{24}In_{28}N_8O_{116}$	$C_{78}H_{78}Fe_6In_{13}O_{48}$	$C_{96}H_{90}Fe_6In_{13}N_{12}O_{42}$
**Formula weight**	10392.2	3611.16	4103.55
**Temperature/K**	293(2)	293(2)	100.00(10)
**Crystal system**	tetragonal	monoclinic	hexagonal
**Space group**	$P4_2/n$	$C2/c$	R-3
**a/Å**	35.1846(4)	32.0443(7)	19.80870(10)
**b/Å**	35.1846(4)	14.9493(3)	19.80870(10)
**c/Å**	15.8530(4)	28.0793(7)	28.7268(2)
**α/°**	90	90	90
**β/°**	90	94.153(2)	90
**γ/°**	90	90	120
**Volume/Å^3**	19625.3(7)	13415.8(5)	9761.79(12)
**Z**	2	4	3
**ρ_{calc}g/cm^3**	1.759	1.788	2.094
**Goodness-of-fit on F^2**	1.013	1.077	1.093
**Final R indexes [I>=2σ (I)]**	R_1 = 0.0646, wR_2 = 0.1765	R_1 = 0.0457, wR_2 = 0.1321	R_1 = 0.0628, wR_2 = 0. 1894
**Final R indexes [all data]**	R_1 = 0.1107, wR_2 = 0.2006	R_1 = 0.0611, wR_2 = 0.1396	R_1 = 0.0676, wR_2 = 0.1921
**CCDC No.**	2281566	2281561	2281558

Table S 3. Crystal data and strutures refinement for **7-9**.

**Identification code**	**InOC-7**	**InOC-8**	**InOC-9**
**Empirical formula**	$C_{92}H_{96}Fe_6In_{13}N_6O_{46}$	$C_{68}H_{56}N_8O_{38}Fe_4In_{13}Cl_2$	$C_{106}H_{86}Fe_6In_{13}O_{52}$
**Formula weight**	3849.5	3380.16	4019.5
**Temperature/K**	100.01(10)	99.99(10)	179.97(10)
**Crystal system**	monoclinic	monoclinic	monoclinic
**Space group**	$I2/a$	$C2/m$	$C2/m$
**a/Å**	27.37206(19)	19.6313(8)	21.2041(3)
**b/Å**	15.00755(12)	22.9290(6)	25.0454(3)
**c/Å**	31.8968(3)	13.3713(4)	13.8544(2)
**α/°**	90	90	90
**β/°**	94.0511(7)	95.854(3)	103.7827(15)
**γ/°**	90	90	90
**Volume/Å^3**	13070.05(17)	5987.4(3)	7145.74(18)

**Z**	4	2	2
**ρ_{calc}g/cm^3**	1.956	1.875	1.868
**Goodness-of-fit on F^2**	1.082	1.05	1.062
**Final R indexes [I>=2σ (I)]**	R_1 = 0.0641, wR_2 = 0.1829	R_1 = 0.0483, wR_2 = 0.1318	R_1 = 0.0576, wR_2 = 0.1622
**Final R indexes [all data]**	R_1 = 0.0685, wR_2 = 0.1872	R_1 = 0.0539, wR_2 = 0.1356	R_1 = 0.0642, wR_2 = 0.1690
**CCDC No.**	2281563	2281559	2281560

Table S 4. Bond Valence Sum (BVS) Calculations for **2**

**Atom**	**charge**	**BVS value Fe^{II}**	**BVS value Fe ^{III}**
Fe1	2	1.899075	2.031825

r_o value of Fe^{II}-O is 1.734Å. r_o value of Fe^{III}-O is 1.765Å. The calculated value of BVS value rounded to the nearest whole number 2.

3. Molecular Structure

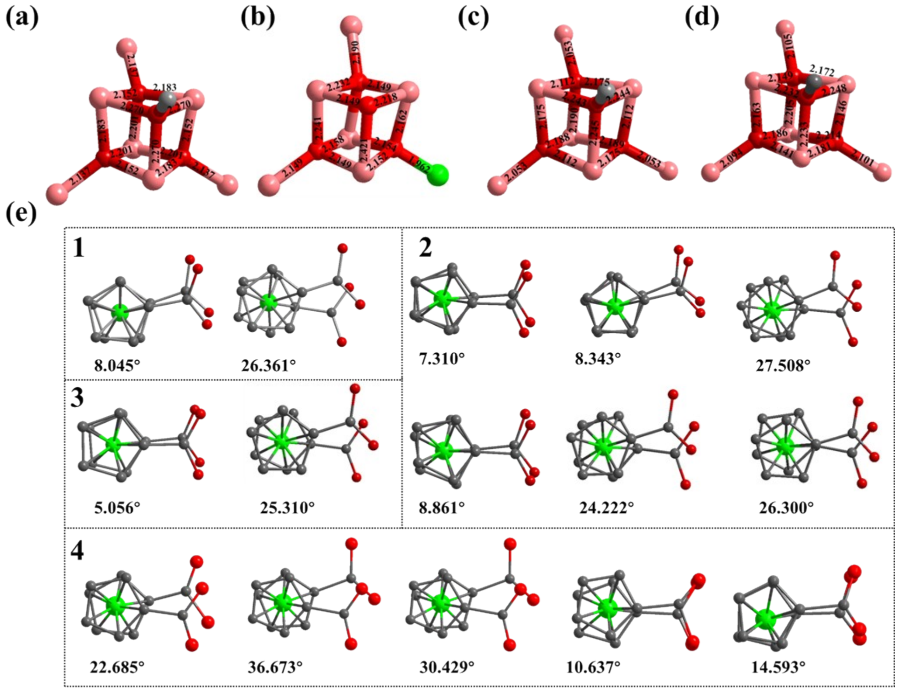

Figure S1. **(**(a, b, c, d)The bond length of In-O and Fe-O in the central cubane cluster cores of compound **1-4**; (e) the observed torsion angles of the $FcDCA^{2-}$ ligands is in compound **1-4.**)

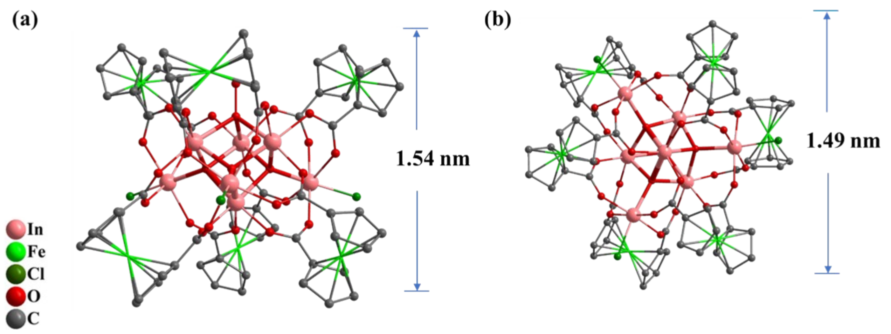

Figure S2. The crystal structure of compound **1**, emphasizing its nano-sized dimensions (top (a) and side (c) views).

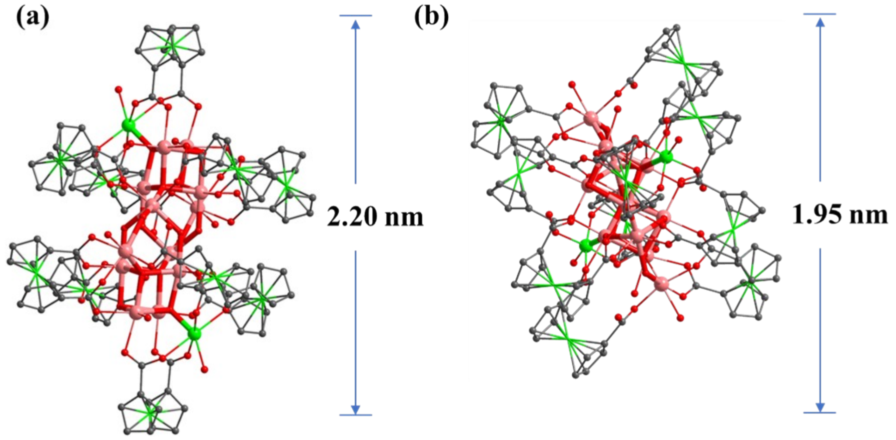

Figure S3. The crystal structure of compound **2**, emphasizing its nano-sized dimensions (top (a) and side (c) views).

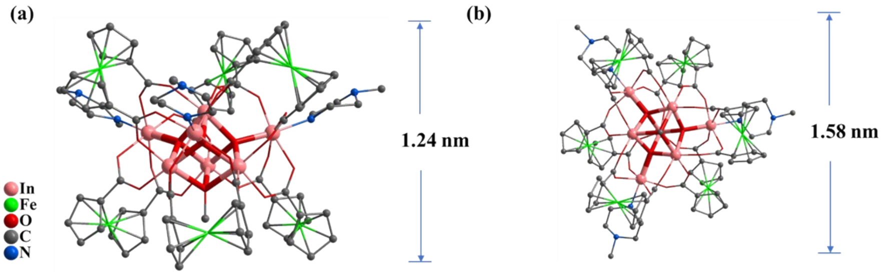

Figure S4. The crystal structure of compound **3**, emphasizing its nano-sized dimensions (top (a) and side (c) views).

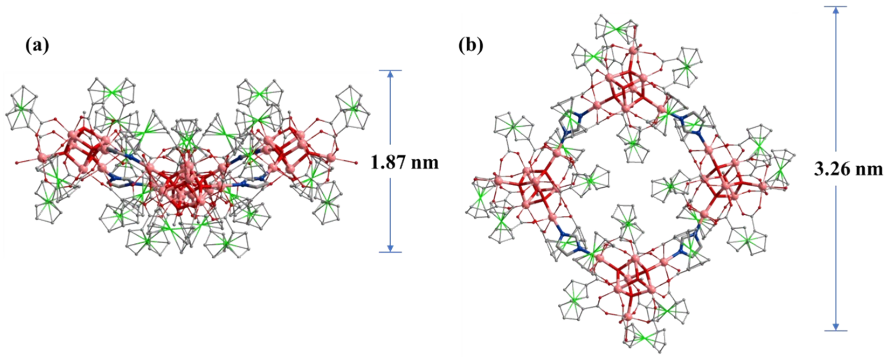

Figure S5. The crystal structure of compound **4**, emphasizing its nano-sized dimensions (top (a) and side (c) views).

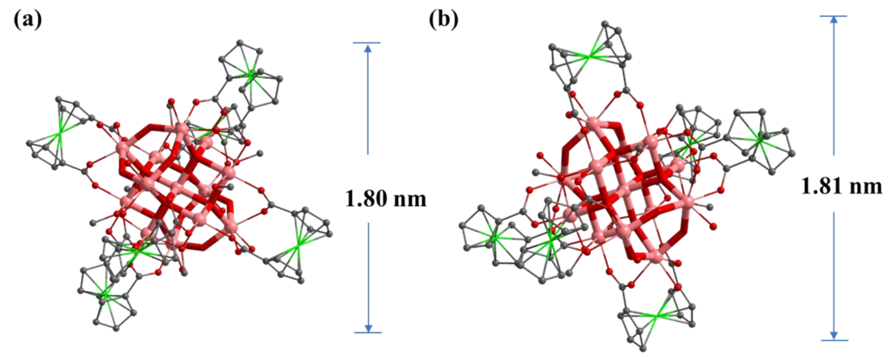

Figure S6. The crystal structure of compound **5**, emphasizing its nano-sized dimensions (top (a) and side (c) views).

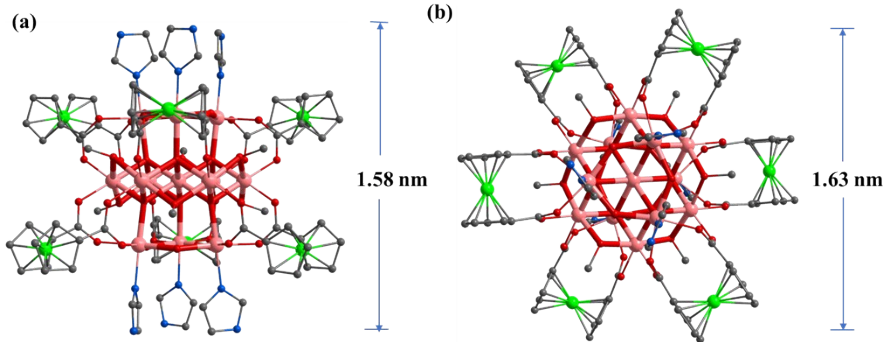

Figure S7. The crystal structure of compound **6**, emphasizing its nano-sized dimensions (top (a) and side (c) views).

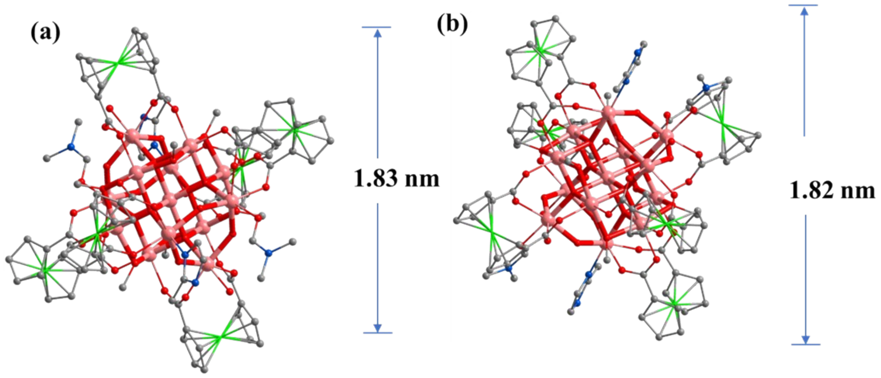

Figure S8. The crystal structure of compound **7**, emphasizing its nano-sized dimensions (top (a) and side (c) views).

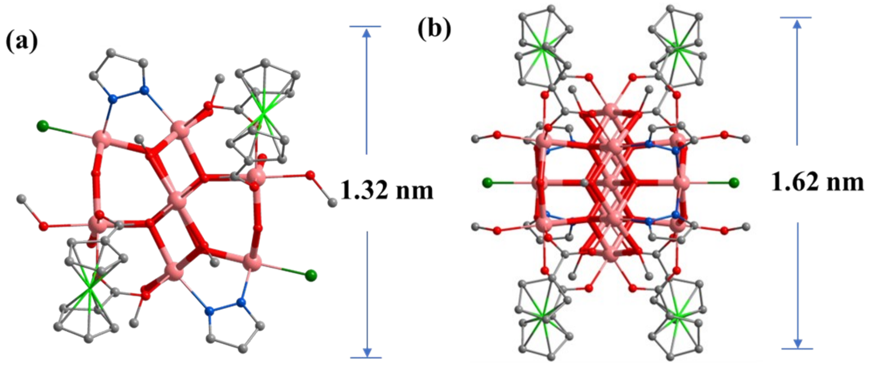

Figure S9. The crystal structure of compound **8**, emphasizing its nano-sized dimensions (top (a) and side (c) views).

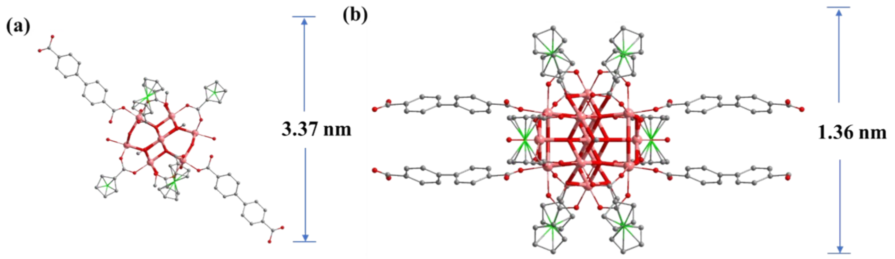

Figure S10. The crystal structure of compound **9**, emphasizing its nano-sized dimensions (top (a) and side (c) views).

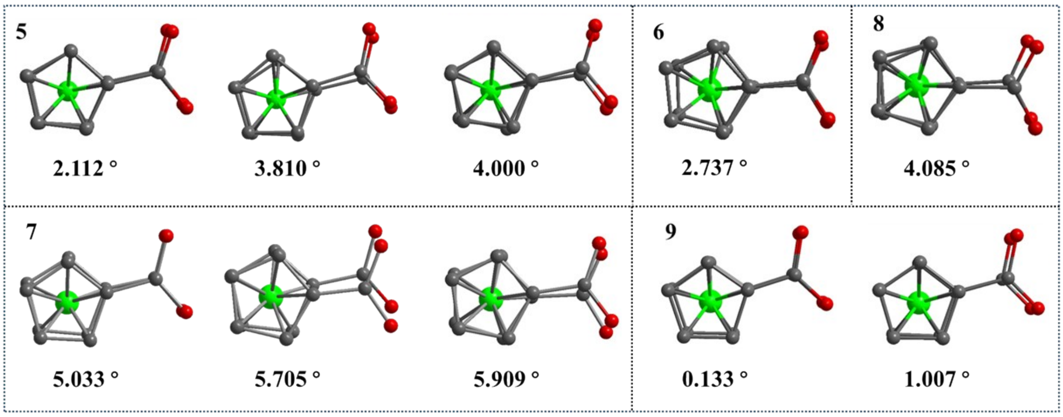

Figure S11. The observed torsion angles of the $FcDCA^{2-}$ ligands is in compound **5-9.**

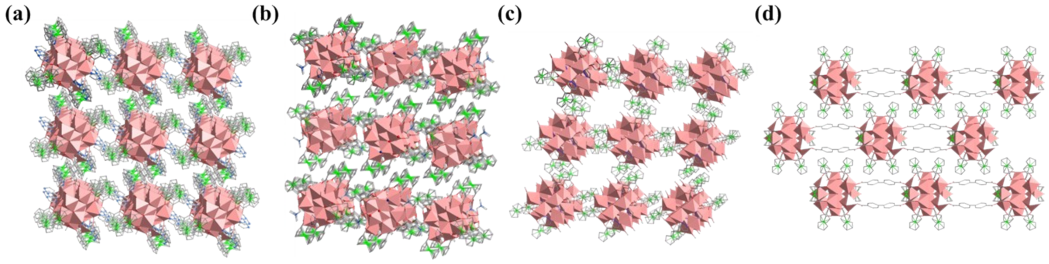

Figure S12. (a) Compound **6** is stacked in AAA form along the cluster c-axis;(b) **7** is stacked along the cluster b-axis;(c) **8** is stacked along the cluster c-axis;(d) **9** is stacked along the cluster c-axis.

4. Characterizations

PXRD analyses

pH stability and solvent stability

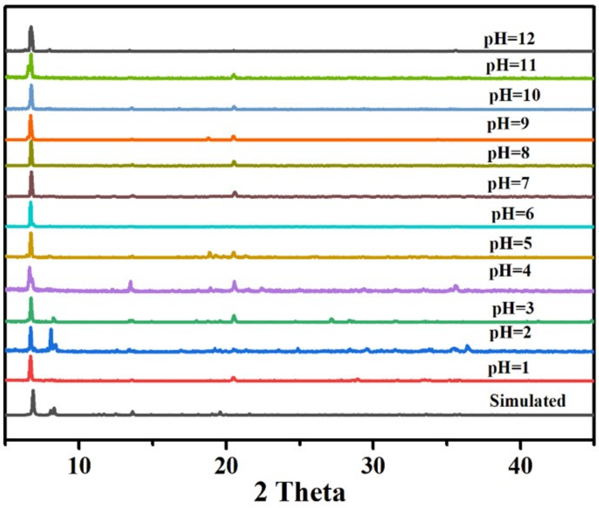

Figure S13. PXRD spectra of **1** in different pH aqueous solution.

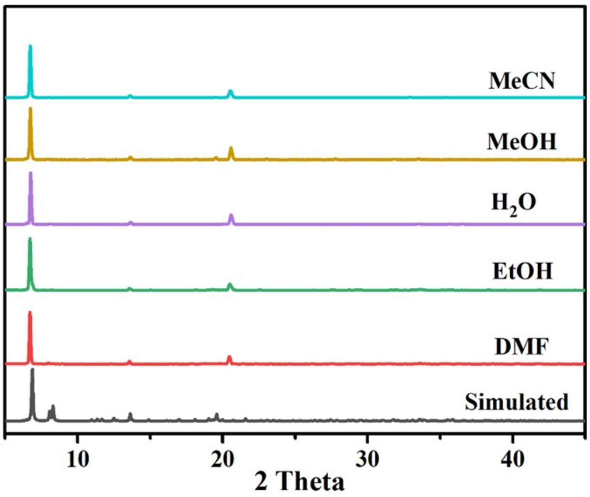

Figure S14. PXRD spectra of **1** in different solutions.

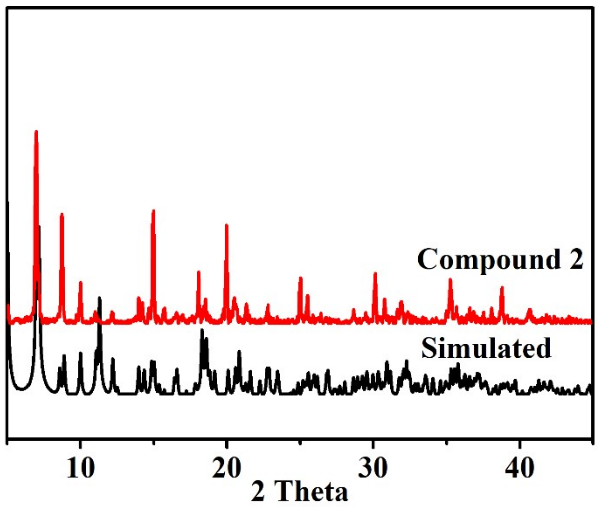

Figure S15. The PXRD patterns of compounds **2**.

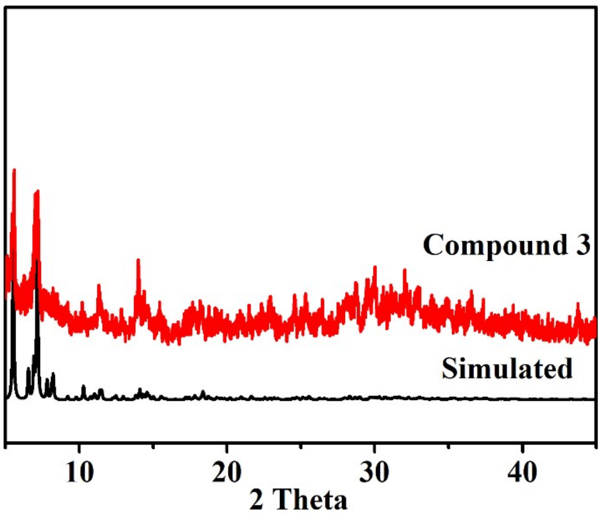

Figure S16. The PXRD patterns of compounds **3**.

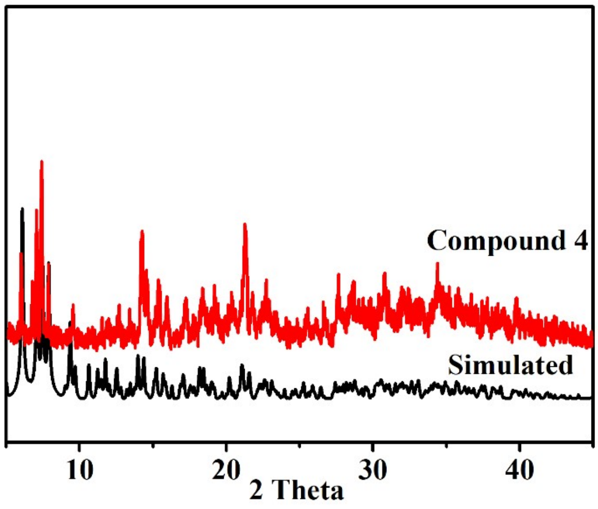

Figure S17. The PXRD patterns of compounds **4**.

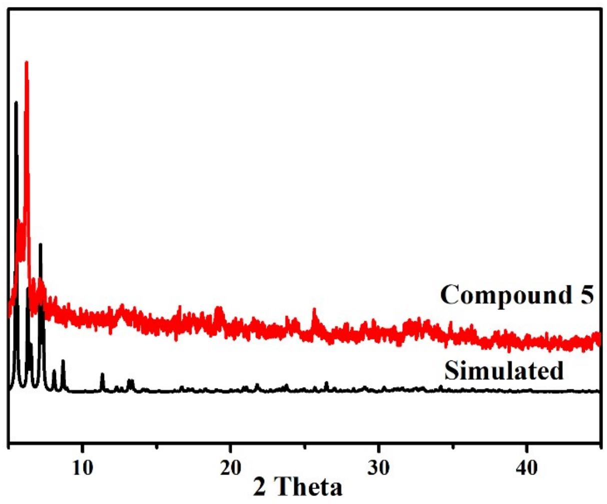

Figure S18. The PXRD patterns of compounds **5**.

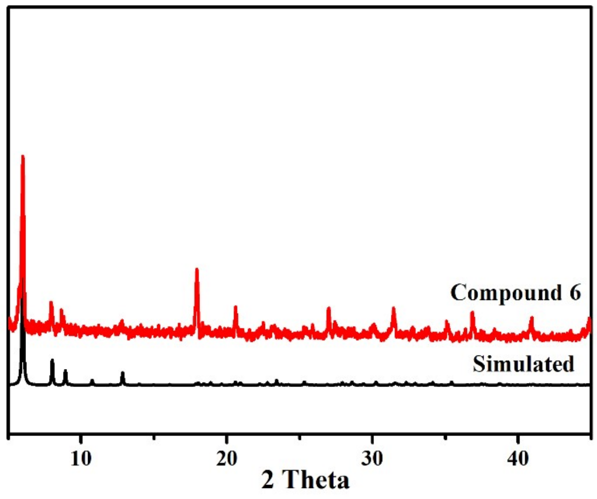

Figure S19. The PXRD patterns of compounds **6**.

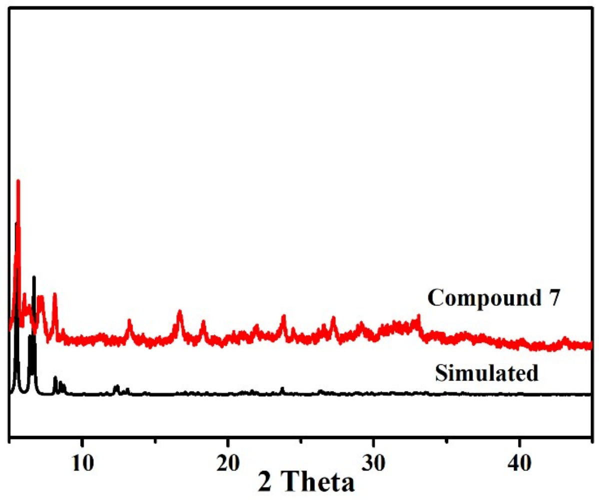

Figure S20. The PXRD patterns of compounds **7**.

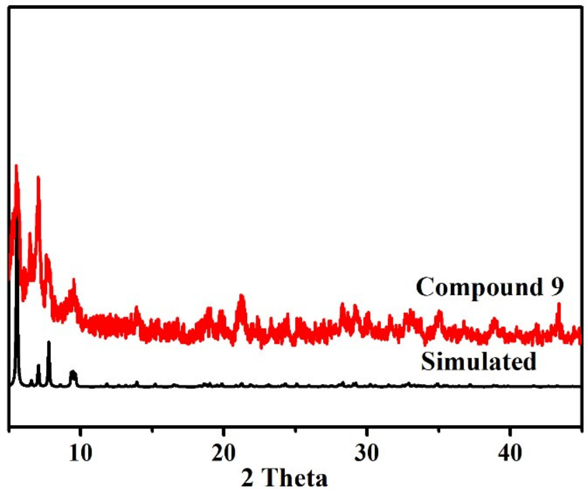

Figure S21. The PXRD patterns of compounds **9**.

Infrared spectrum analysis

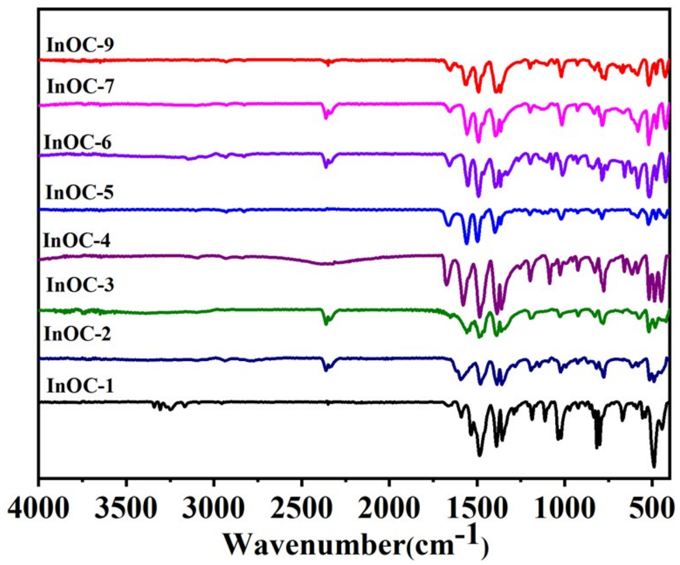

Figure S22. Infrared (IR) spectroscopy of **1** to **7** and **9**.

ICP analyses

Table S 5. ICP analyses of **1**, **2**, **4** and **5** (The total amount of In and Fe is normalized to 100%).

Compound	In		Fe	
	Calculated (%)	Found (%)	Calculated (%)	Found (%)
**1**	70.58	69.53	29.42	30.47
**2**	67.27	68.03	32.73	31.97
**4**	70.58	70.51	29.42	29.49
**5**	81.67	80.12	18.33	19.88

TGA

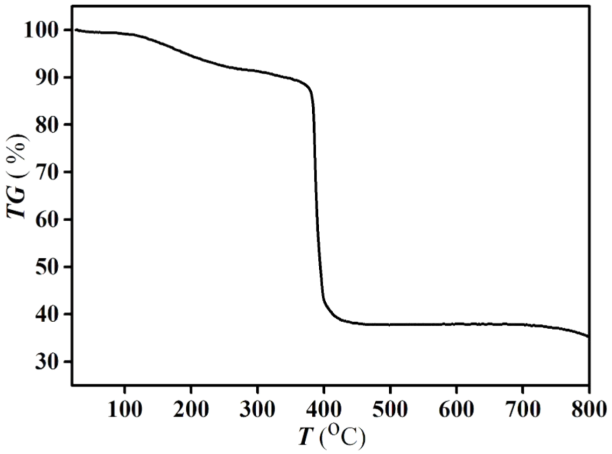

Figure S23. The TG plot of as synthesized **1**.

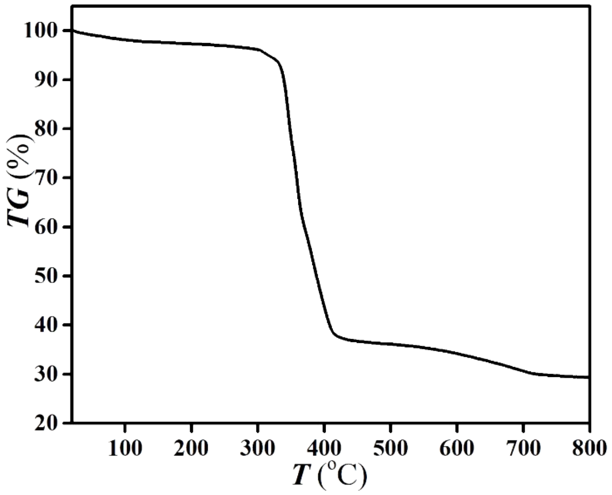

Figure S24. The TG plot of as synthesized **2**.

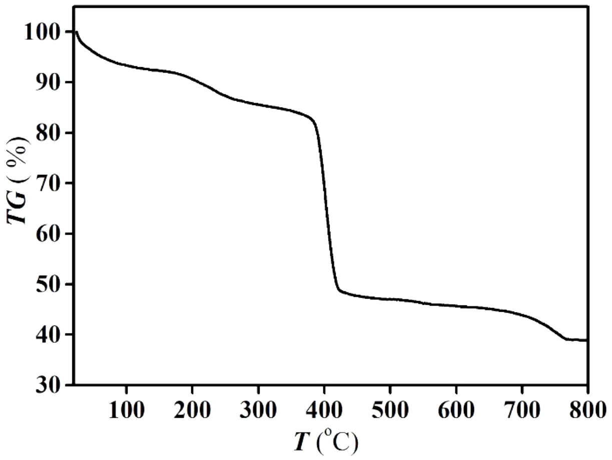

Figure S25. The TG plot of as synthesized **3**.

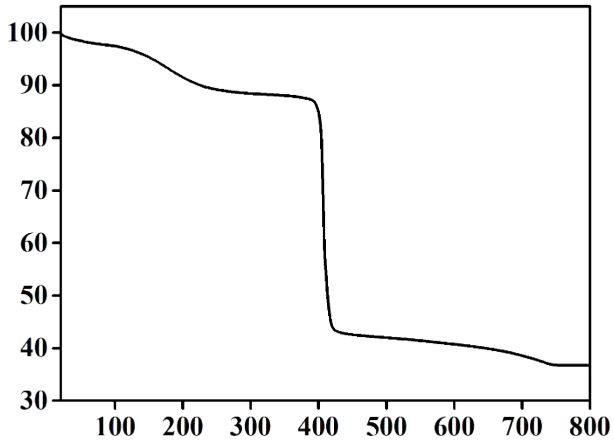

Figure S26. The TG plot of as synthesized **4**.

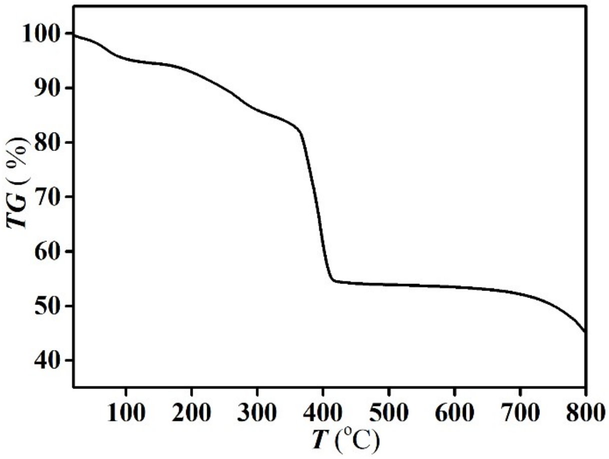

Figure S27. The TG plot of as synthesized **5**.

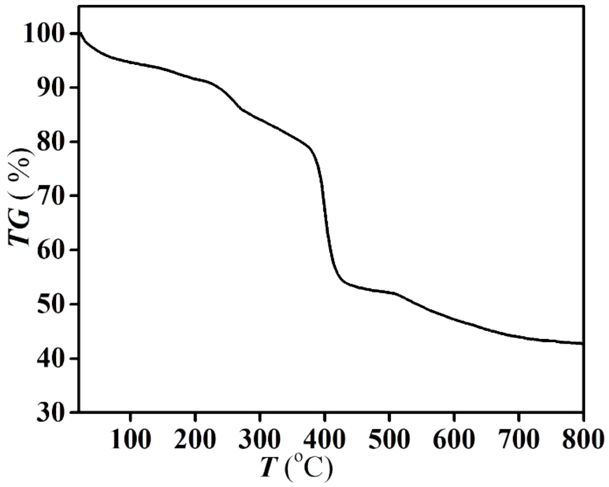

Figure S28. The TG plot of as synthesized **6**.

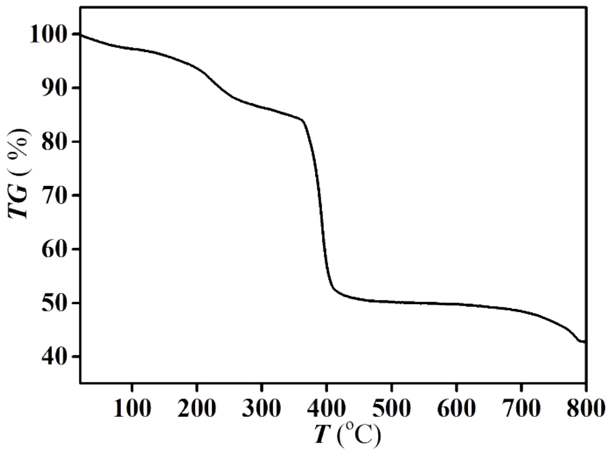

Figure S29. The TG plot of as synthesized **7**.

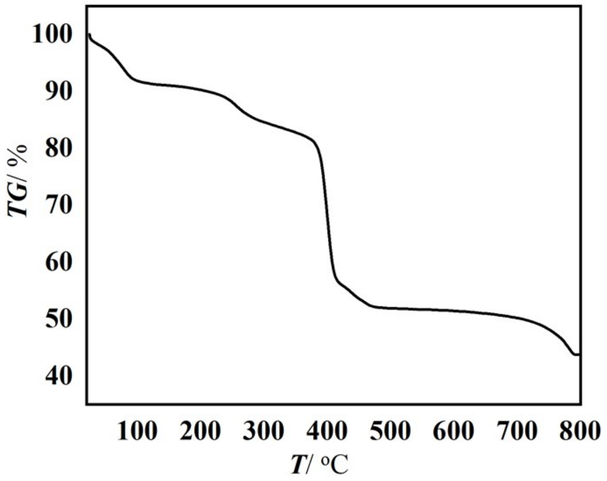

Figure S30. The TG plot of as synthesized **9**.

UV-Vis spectra

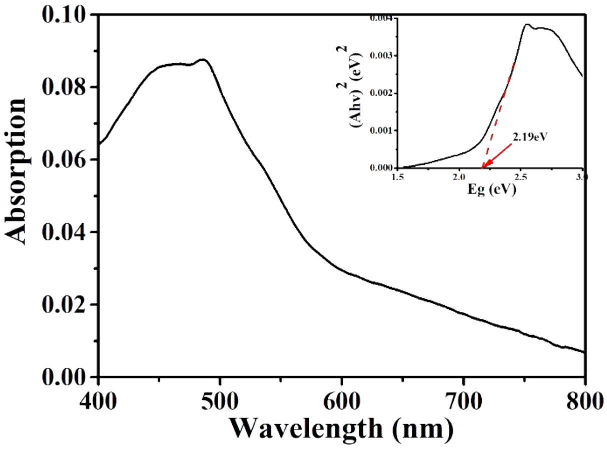

Figure S31. UV-Vis spectra of H_2FcDCA and the band gap is 2.19 eV.

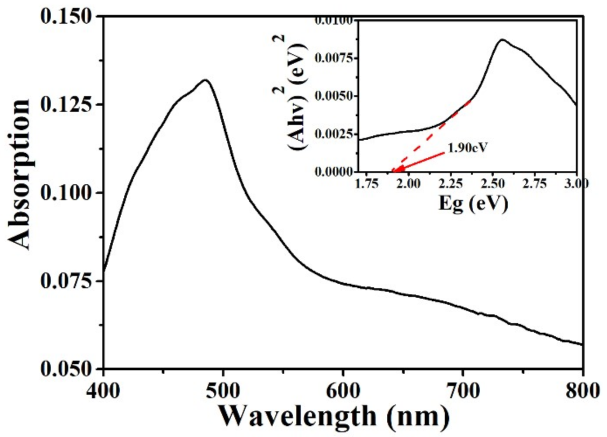

Figure S32. UV-Vis spectra of **1** and the band gap is 1.90 eV.

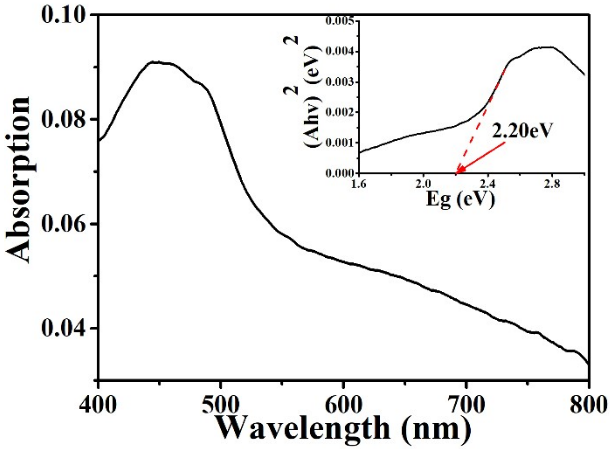

Figure S 33. UV-Vis spectra of **2** and the band gap is 2.20 eV.

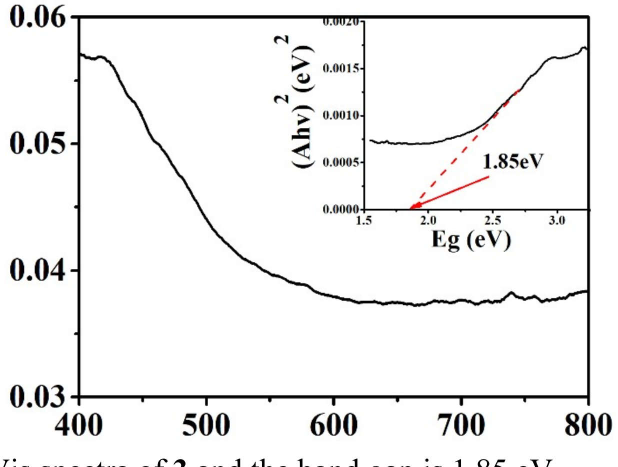

Figure S 34. UV-Vis spectra of **3** and the band gap is 1.85 eV.

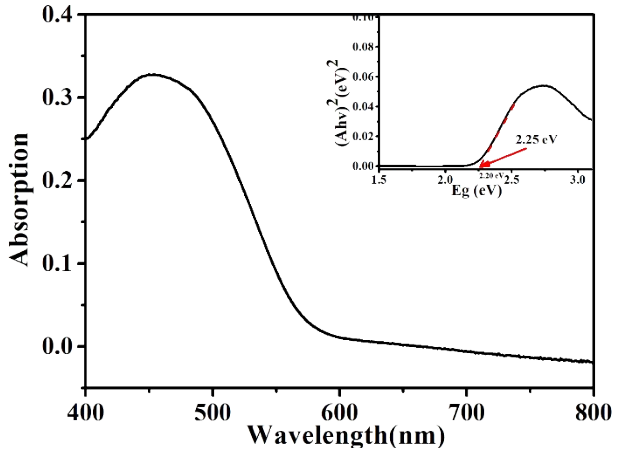

Figure S 35. UV-Vis spectra of **4** and the band gap is 2.25 eV.

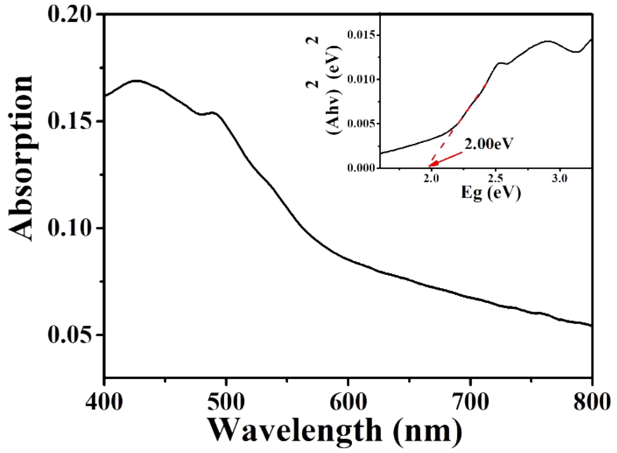

Figure S 36. UV-Vis spectra of **5** and the band gap is 2.00 eV.

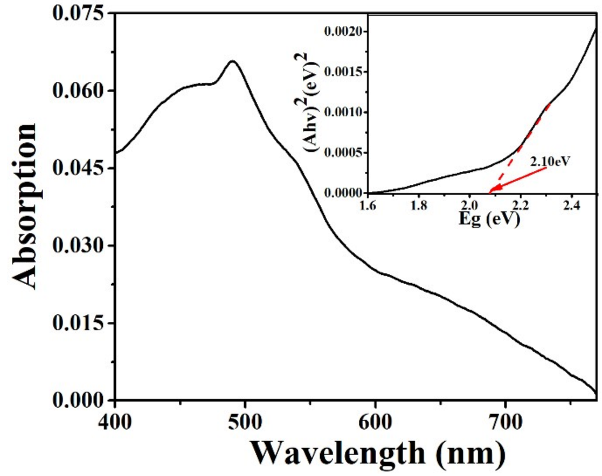

Figure S 37. UV-Vis spectra of **6** and the band gap is 2.10 eV.

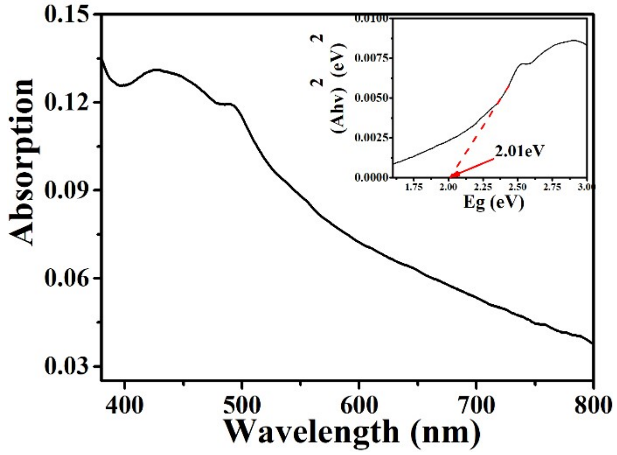

Figure S 38. UV-Vis spectra of **7** and the band gap is 2.01 eV.

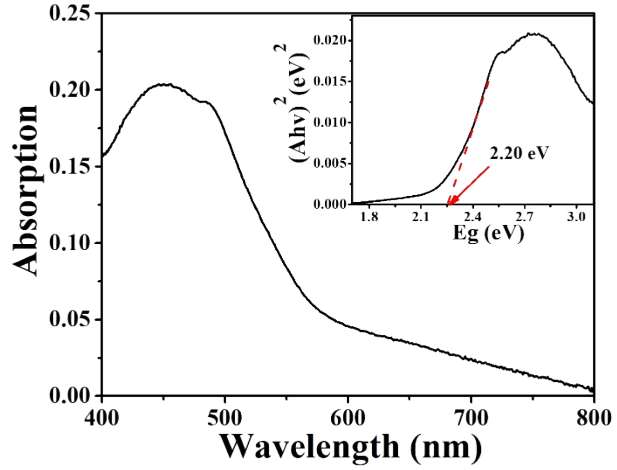

Figure S 39. UV-Vis spectra of **9** and the band gap is 2.20 eV.

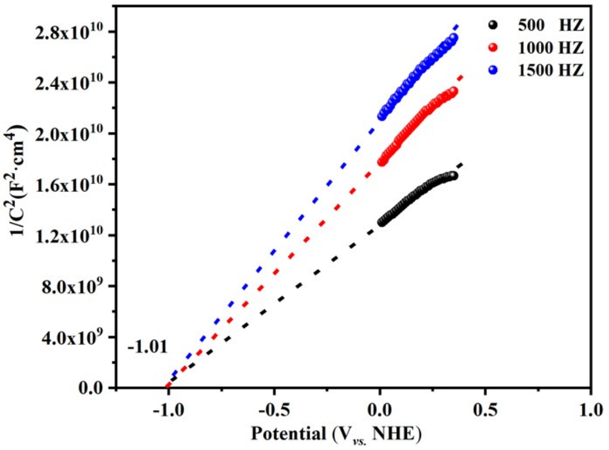

Figure S 40. Mott Schottk plots of **1**

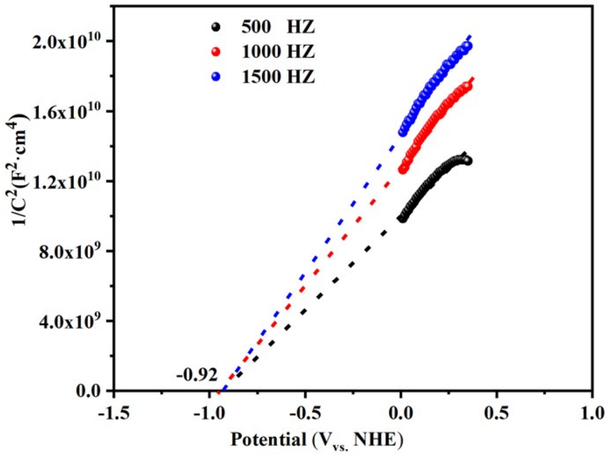

Figure S 41. Mott Schottk plots of **3**

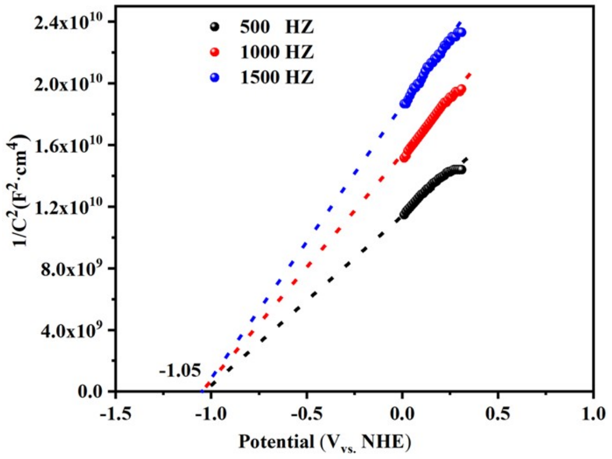

Figure S 42. Mott Schottk plots of **4**

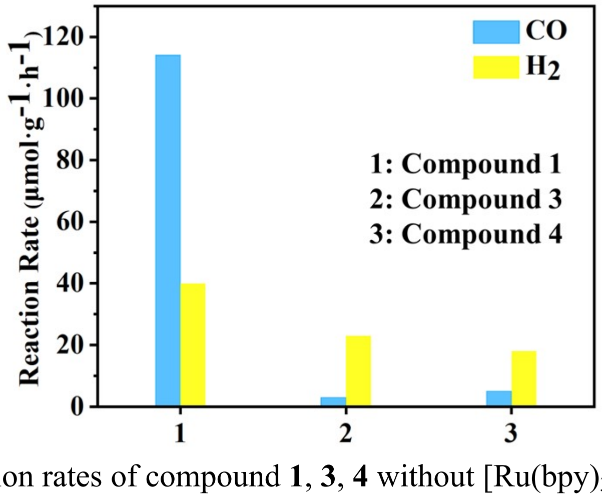

Figure S 43. Reaction rates of compound **1**, **3**, **4** without $[Ru(bpy)_3]Cl_2{\cdot}6H_2O$.
